# Immune cell-specific genetic architecture of Alzheimer’s disease revealed by multi-omics analysis for therapeutic target discovery and prioritization

**DOI:** 10.1038/s41398-026-04199-9

**Published:** 2026-06-23

**Authors:** Tian-long Gao, Shan Geng, Jia Chen, Liu Yang, Yun-juan Nie, Haitao Yu, Gao-shang Chai

**Affiliations:** 1 Department of Fundamental Medicine, Wuxi School of Medicine, Jiangnan University, Wuxi, Jiangsu 214122 P. R. China; 2 MOE Medical Basic Research Innovation Center for Gut Microbiota and Chronic Diseases, School of Medicine, Jiangnan University, Wuxi, Jiangsu China; 3 Department of Pathology, Affiliated Hospital of Jiangnan University, Wuxi, 214122 China

**Keywords:** Pharmacogenomics, Molecular neuroscience

## Abstract

Alzheimer’s disease (AD) is a multifactorial neurodegenerative condition in which accumulating genetic and molecular evidence implicates dysregulation of peripheral immune processes in disease pathogenesis. Nevertheless, the contribution of distinct peripheral immune cell subsets and associated gene regulatory landscapes to AD risk remains incompletely defined. To address this gap, we integrated single-cell expression quantitative trait loci (sc‑eQTL) data from the OneK1K cohort with AD GWAS summary statistics. We systematically interrogated immune cell-specific genes for their contributions to AD risk by integrating genetic causal inference with Bayesian colocalization analyses, and identified 24 eGenes that passed both the MR significance threshold (*P* < 0.05) and the criterion for strong shared genetic signals (PP.H4 > 0.8). Notable candidates included GATS, HLA-DOB, HLA-DQA1, PM20D1, and others, with each gene demonstrating a cell-type-specific association restricted to its corresponding immune cell type, such as monocytes, CD8^ + ^T cells, or B cells. Independent peripheral blood single-cell transcriptomic data further supported disease-associated shifts in cell-type-specific expression patterns in AD. Phenome-wide association studies (PheWAS) indicated limited associations with off-target traits, indicating a favorable safety profile for therapeutic intervention, with the exceptions of B4GALNT3, PM20D1, and CNN2. Integration of immune gene targets with pharmacological databases yielded three candidate compound, including NSC321521 (targeting HLA-DQA1), phenoxybenzamine (targeting GSTP1), and rimexolone (targeting BIN1). Among these compounds, Predicted blood-brain barrier permeability was observed only for phenoxybenzamine and rimexolone, with docking studies indicating stable interactions, such as those between NSC321521 and HLA-DQA1, phenoxybenzamine and GSTP1, and rimexolone and BIN1. This integrative approach highlights key immune‑cell‑specific genes involved in AD and proposes repurposable drugs with central nervous system potential, paving the way for more targeted immunomodulatory strategies in AD.

## Introduction

Alzheimer’s disease (AD) is a common and progressive neurodegenerative disorder — the most frequent cause of dementia in older adults, and represents a major and growing global public-health challenge due to its substantial prevalence, rising incidence, and profound societal burden [1, 2]. Key neuropathological markers of AD comprise extracellular β-amyloid (Aβ) plaque deposition and the intracellular aggregation of hyperphosphorylated tau into neurofibrillary tangles; these are accompanied by widespread synaptic and neuronal loss, as well as white-matter and synapse degeneration [3, 4].

Increasing data indicate that immune mechanisms are involved in AD. Postmortem examinations reveal glial activation (microglia and astrocytes) and recruitment of peripheral immune cells to AD-affected brain regions [5–8]. Moreover, peripheral cytokine dysregulation and immune cell remodeling have been observed in AD [8–11]. These observations have motivated investigations into immunomodulatory strategies, and genetic analyses further highlight immune-related loci as contributors to AD susceptibility [12, 13]. However, the detailed roles of peripheral immune cells and their regulatory mechanisms in AD remain unclear, and elucidating these pathways may guide the development of targeted and safer therapeutic strategies.

Leveraging human genetic evidence enables the identification of causally relevant genes, providing a rational basis for therapeutic target selection and enhancing the prospects of clinical trial success [14–16]. Findings from genome-wide association studies have mapped several independent genetic loci for AD, implicating key molecular pathways, including amyloid-β processing, tau pathology, lipid metabolism, and immune response [12]. However, elucidating effector genes and their corresponding tissues or cell types remains challenging due to the complexity of linkage disequilibrium (LD) and regulatory mechanisms. Mendelian randomization (MR), which leverages genetic variants as instrumental variables (IVs), enables causal inference between gene expression and disease [17]. Several studies have applied genetic approaches to identify druggable AD targets from GWAS, including transcriptome-wide association studies (TWAS), proteome-wide MR, and multi-omics integration [18, 19]. While informative for drug target discovery, approaches lacking cell-resolved genetic regulation may fail to inform disease mechanisms or guide precise therapeutic strategies.

Recent advances in single-cell expression quantitative trait locus (sc-eQTL) analysis allow dissection of gene regulatory networks within specific immune cell populations [20–23], offering enhanced resolution for identifying causal genes and therapeutic targets in complex disorders such as AD.

**Fig. 1:**
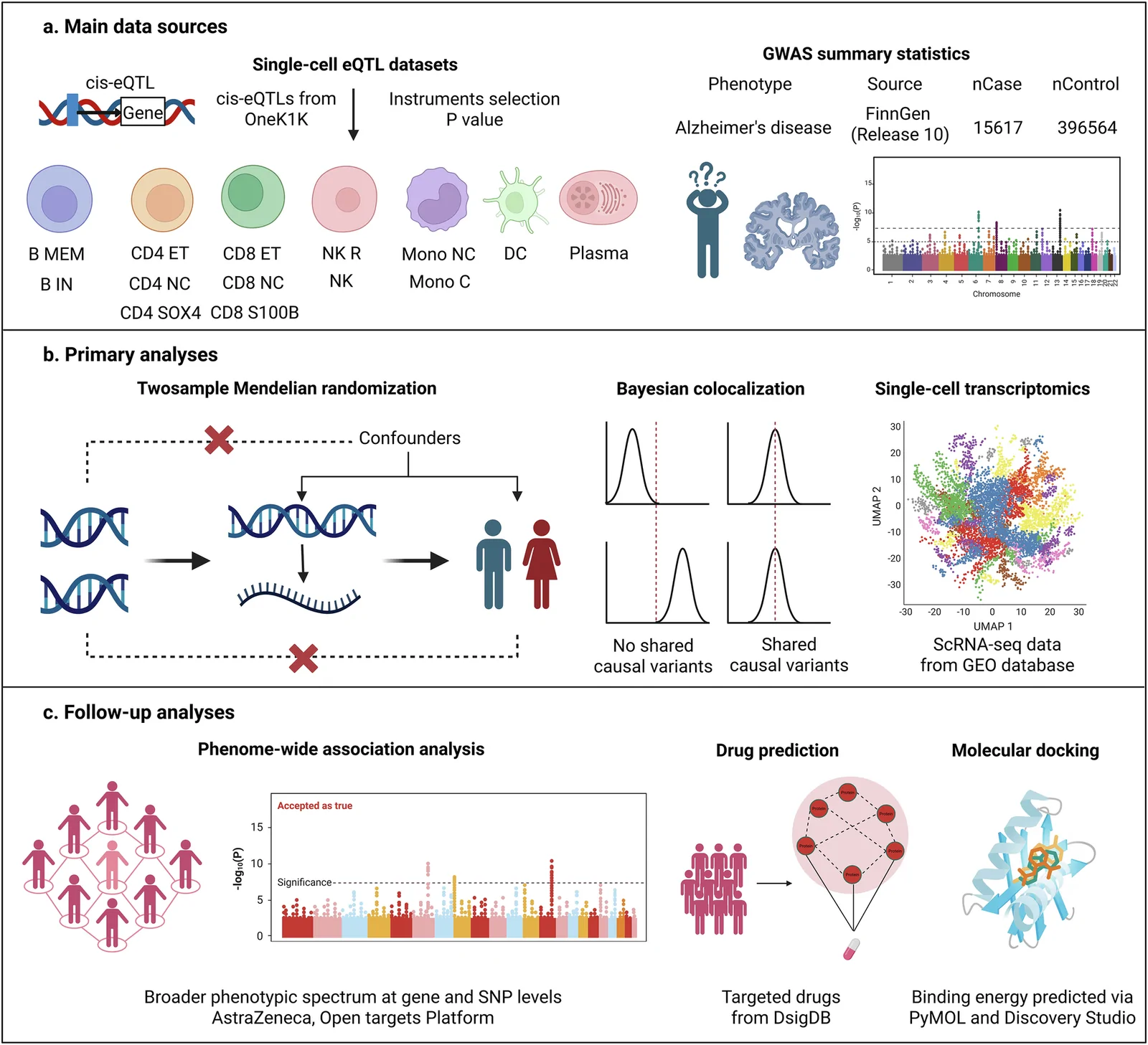
Overview of study design and analytical workflow. **a** Input data: single-cell eQTL data from 14 peripheral immune cell types (OneK1K cohort) and GWAS summary statistics of Alzheimer’s disease. **b** Primary analyses: sc-eQTL-based MR analysis to infer cell type–specific causal associations, Bayesian colocalization to identify shared causal variants, and single-cell RNA sequencing to characterize peripheral immune cell composition. **c** Follow-up analyses: phenome-wide association studies (PheWAS) to evaluate pleiotropic effects and exclude genes with predicted off-target consequences, candidate compound prediction through drug signature enrichment (DSigDB), and molecular docking simulations to assess potential binding with AD-related targets.

Integrating sc-eQTL with AD GWAS, we examined immune-cell–specific gene expression for potential causal effects on disease risk, employing MR and Bayesian colocalization to inform potential therapeutic strategies. To reduce the likelihood of off-target effects, candidate genes were further refined using pleiotropy information derived from PheWAS, excluding those with predicted associations across non-relevant organ systems. The remaining targets were then subjected to drug signature enrichment analyses and structure-based molecular docking to identify putative therapeutic compounds. Collectively, this integrative framework elucidates immune-mediated mechanisms underlying AD susceptibility to enable targeted drug development along defined immune pathways (Fig. 1).

## Materials and methods

### Study design

The analytical strategy employed in this study is summarized in Fig. 1. We established an integrative framework to assess whether immune-cell–specific gene regulatory variation contributes causally to AD risk. To accomplish this, sc-eQTL datasets were combined with summary-level genome-wide association statistics for AD, enabling the construction of a cell-type–resolved causal inference architecture. The framework comprised four major analytical stages: (i) derivation of genetic instruments from sc-eQTL profiles; (ii) Preliminary analyses included MR to estimate causal effects, Bayesian colocalization to identify shared causal variants, and single-cell transcriptomic analyses to validate cell-type-specific expression patterns; and (iii) downstream functional follow-up, including phenome-wide association analyses of instrumental variants and small-molecule prioritization through drug enrichment and molecular docking. MR estimation was performed under the standard instrumental-variable paradigm [17]. This paradigm requires that the selected variants represent robust proxies for the exposure, that they exhibit no association with confounding factors, and that their influence on the outcome operates exclusively through the exposure. To ensure adherence to these criteria, we applied harmonization procedures to standardize effect alleles and genomic annotations, and we implemented stringent filters to remove instruments with characteristics inconsistent with core MR assumptions.

### Exposure data and instrument selection

Discovery sc-eQTL summary statistics were obtained from the OneK1K resource, with independent single-cell RNA-seq datasets used for replication. The OneK1K dataset includes 1,267,758 peripheral blood mononuclear cells profiled from 982 healthy donors of Northern European ancestry. On the basis of transcriptomic signatures, 14 immune cell types were delineated. For each gene–cell-type combination, cis-eQTLs were mapped within ±1000 kb of gene boundaries, and summary statistics were available for the lead variants. Only associations surpassing *P* < 1 × 10⁻⁵ were retained to ensure the use of robust and biologically meaningful instruments and to minimize the inclusion of unstable or weak regulatory signals. This threshold aligns with the false-discovery-rate–corrected significance level (FDR < 0.05) observed in two preceding studies [24, 25], thereby maintaining consistency in statistical stringency across analytical frameworks.

### Outcome data

To evaluate genetic liability to AD, we used summary statistics from the FinnGen Release 10 (R10) genome-wide association study. This dataset comprises 15,617 clinically diagnosed AD cases and 396,564 population controls, all predominantly of Finnish and broader European ancestry. FinnGen represents one of the largest contemporary resources for AD genetics, offering high statistical power for variant discovery [26]. Previous functional annotations of FinnGen-derived risk loci implicate biological pathways central to AD pathogenesis, including amyloid processing, tau-related mechanisms, neuroinflammation, and lipid metabolism, thereby supporting the suitability of these data for downstream MR analyses [27].

### MR using Sc-eQTL data

Using the TwoSampleMR package (v0.6.15), we conducted two-sample MR to assess the causal role of cell-type–resolved immune gene expression in AD. Causal estimates were computed according to the availability of valid instruments: eGenes represented by a single independent variant were evaluated using the Wald ratio, whereas those supported by multiple independent instruments were analyzed using the inverse-variance weighted estimator. Statistical significance was determined on the basis of MR *P* values, and only associations surpassing the predefined significance criterion were advanced to subsequent analyses. Due to the cell-type-restricted nature of single-cell regulatory signals, single-SNP instruments were frequently utilized. While multi-variant pleiotropy tests are not applicable here, we addressed potential horizontal pleiotropy by requiring stringent Bayesian colocalization (PP.H4 > 0.80). This ensures that a single causal variant drives both sc-eQTL and AD risk, minimizing the likelihood of confounding effects from neighboring genes in linkage disequilibrium. Genes meeting this threshold were subjected to downstream evaluation, including Bayesian colocalization, protein-level evidence assessment, and in silico druggability analyses. This strategy enabled the prioritization of eGenes in specific immune cell types that may play causal roles in AD.

### Colocalization study

To determine whether the same genetic variants influenced both gene expression and AD risk, we performed colocalization analysis using the coloc R package (v5.2.3). For each candidate eGene, posterior probabilities were estimated for five alternative causal configurations, with PP.H4 reflecting the likelihood of a common causal variant underlying both traits. Genes were subsequently stratified according to colocalization strength, with PP.H4 > 0.80 indicating high-confidence evidence and values between 0.50 and 0.80 denoting moderate support. Only high-confidence eGenes were retained for downstream functional interpretation and drug-target prioritization, thereby limiting spurious findings attributable to linkage disequilibrium or random signal overlap and ensuring robust immune-cell-specific inferences.

### Pre-processing and annotation of peripheral blood scRNA-seq data

We analyzed scRNA-seq data from a public resource (GSE181279) with two controls and three AD patients, processing raw counts and metadata in Seurat (v5.1.0) within R (v4.4.0). Quality control retained cells with fewer than 10% mitochondrial gene expression and between 200 and 4000 detected genes. Hemoglobin gene expression was calculated to assess potential blood contamination. Gene expression data were log-normalized, and the top 2000 highly variable genes were identified for downstream analysis. To mitigate technical and donor-specific confounders, we implemented a robust integration pipeline. Batch effects across the five donors were addressed using Harmony-based integration, which projects cells into a shared embedding space while preserving biological variation. The data were scaled to regress out effects of total detected genes and mitochondrial gene content. Dimensionality reduction was performed using principal component analysis (PCA), followed by t-distributed stochastic neighbor embedding (t-SNE) for visualization of cellular heterogeneity. A shared nearest-neighbor graph was constructed, and graph-based clustering was applied at multiple resolutions to identify stable cell clusters. Cluster stability across resolutions was evaluated, and a final resolution was selected for downstream analyses.

### Cross-resource phenome-wide association analysis

To characterize the broader phenotypic spectrum and safety implications of prioritized targets, we conducted phenome-wide association analyses using publicly available databases. Gene-level associations were queried in the AstraZeneca PheWAS Portal (https://azphewas.com), which catalogs rare-variant–enriched trait associations from the UK Biobank. Phenotype associations for each eGene were compiled, with those meeting *P* < 1 × 10⁻⁸ classified as significant and those with *P*-values from 1 × 10⁻⁸ to 1 × 10⁻⁶ considered suggestive. This approach enabled systematic evaluation of immune-related and non-immune phenotypic links in the general population. In parallel, SNP-level PheWAS was performed for instrumental variants used in the MR analyses. Data on variant–phenotype associations were obtained from the publicly available Open Targets Platform (https://platform.opentargets.org), which aggregates GWAS credible-set evidence across multiple cohorts. Significant associations, determined after Bonferroni correction, were grouped by immune and non-immune phenotypes to facilitate biological interpretation and consideration of on-target safety.

### DSigDB-based drug prediction

We explored potential therapeutics for prioritized AD eGenes employing DSigDB through the Enrichr. This resource maps bioactive compounds in relation to gene expression patterns, enabling the identification of compounds that could affect the prioritized eGenes. Prioritized eGenes from MR and colocalization were analyzed for enrichment, with scores determined by overlap with drug-associated profiles. Higher scores indicate stronger associations, resulting in a ranked list of compounds suitable for deeper analysis as targeted modulators of AD-associated immune genes (Table S10).

### Assessment of blood-brain barrier (BBB) penetration

For compounds targeting the prioritized AD-linked eGenes, their capacity to traverse the BBB was evaluated, which is an essential factor for central nervous system efficacy. SMILES representations collected from PubChem were analyzed in SwissADME (http://www.swissadme.ch), which uses physicochemical and predictive criteria to classify compounds as BBB permeable or non-permeable (Table S12).

### Structure-based molecular docking

3D structures for each ligand were collected from PubChem (https://pubchem.ncbi.nlm.nih.gov/) in SDF format. Receptor protein structures were predicted using AlphaFold (https://www.alphafold.ebi.ac.uk/), and the highest-confidence models were selected for downstream analyses. Protein structures were prepared in PyMOL (v2.6.0) by removing water molecules and adding hydrogens, and then saved as PDB files for subsequent docking analyses. Molecular docking was performed using the Auto Blind Docking function in CB-Dock2 (https://cadd.labshare.cn/cb-dock2/php/index.php). For each ligand, 10 rigid conformation searches were conducted, and the binding pose with the highest binding affinity was selected. The resulting protein-ligand complex was downloaded in PDB format for subsequent analyses, and PyMOL (v2.6.0) and Discovery Studio 2019 were used to examine and depict the binding interactions.

### GWAS summary statistics

GWAS summary statistics for AD were obtained from FinnGen Release 10, comprising 15,617 clinically diagnosed cases and 396,564 population-based controls of European ancestry. sc-eQTL data integrating genotyping and scRNA-seq were obtained from the OneK1K cohort, encompassing 1.27 million peripheral blood mononuclear cells from 982 healthy donors. Cell-type-stratified cis-eQTL summary statistics for 14 immune cell types were downloaded from the OneK1K repository, with raw data available via GEO (GSE196830) for replication and quality control.

## Results

### Identification of genetic instruments for immune cell-specific eGenes

**Fig. 2:**
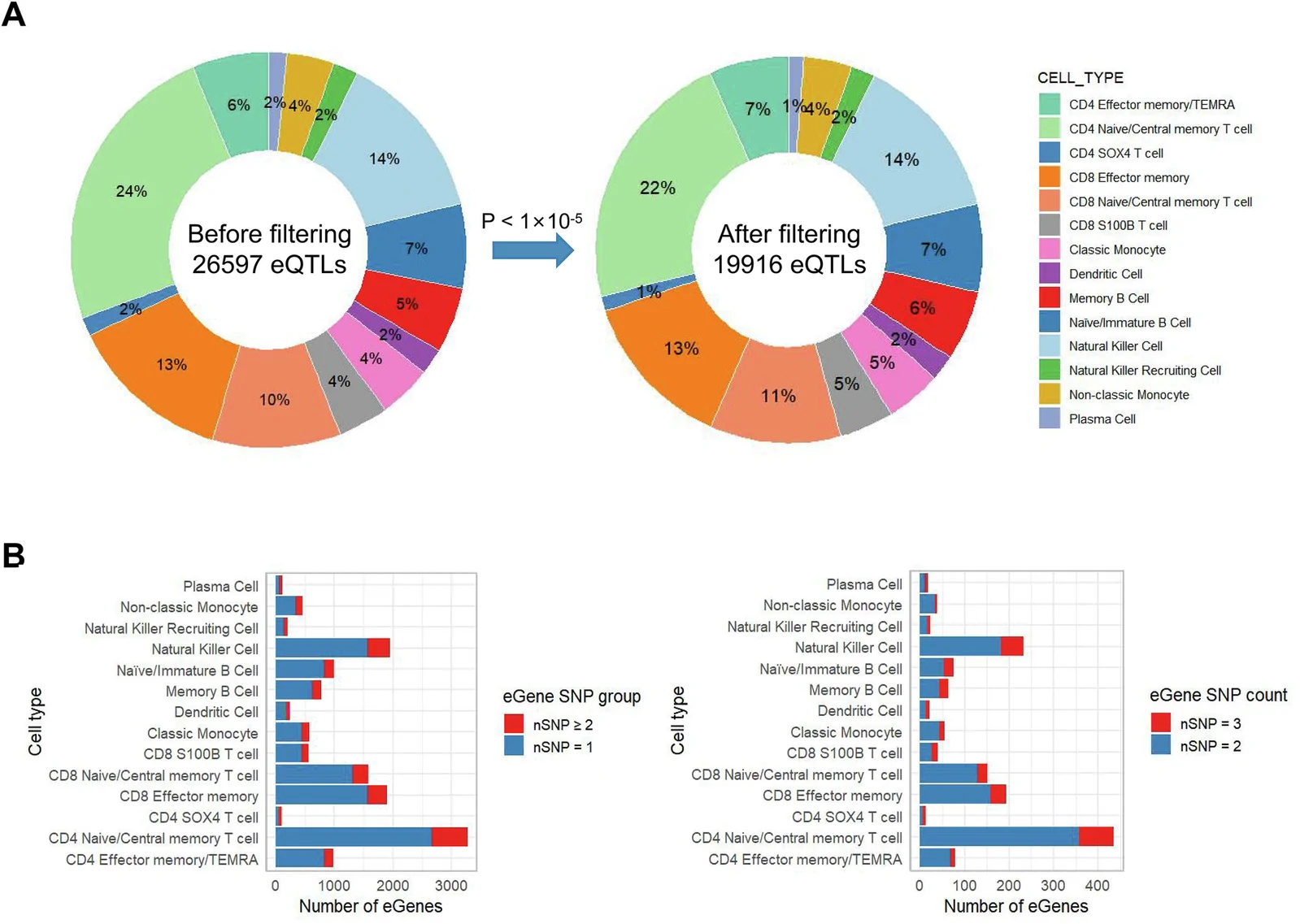
Identification of Genetic Instruments for Immune Cell-Specific eGenes. **A** Pie charts showing the distribution of eGenes across immune cell types before (26,597 eGenes) and after (19,916 eGenes) *P* value filtering. **B** Bar plots depicting the number of eGenes categorized by the number of independent SNPs (nSNP = 1), and the distribution of eGenes with multiple independent SNPs (nSNP = 2, or 3).

To construct robust genetic instruments for MR, cis-eQTLs were assessed across various immune cell types, identifying 26,597 eGenes specific to 14 immune subsets within the OneK1K cohort. At this initial screening stage, LD-based pruning was omitted as sc-eQTL signals are typically dominated by a single lead variant. For each gene–cell-type combination, cis-eQTLs were mapped within ±1000 kb of gene boundaries, and only associations surpassing a significance threshold of *P* < 1 × 10⁻⁵ were retained (Fig. 2A). Instead, we addressed LD-driven confounding at the locus level by requiring subsequent Bayesian colocalization (PP.H4 > 0.80). This ensures that the same causal variant drives both gene expression and AD risk, effectively distinguishing true regulatory effects from LD-linked associations while mitigating weak instrument bias and horizontal pleiotropy. The selection process further stratified eGenes according to the number of independent SNPs serving as genetic instruments. The majority of eGenes were instrumented by a single SNP, thereby minimizing the inclusion of weak or unstable instruments that could introduce bias into Mendelian randomization analyses. In contrast, a smaller subset of eGenes was supported by multiple independent SNPs, providing increased robustness for causal effect estimation in downstream analyses (Fig. 2B). Potential confounding due to LD was addressed in downstream analyses rather than during initial instrument selection. Specifically, candidate eGenes were included in downstream causal inference analyses only when strong colocalization between sc-eQTL and disease-associated GWAS signals was observed, corresponding to a posterior probability for a shared causal variant exceeding 80% (H4 > 0.80). This stringent colocalization criterion effectively mitigates bias arising from correlated variants within LD blocks.

### Causal roles of immune cell-targeted gene expression in AD

**Fig. 3:**
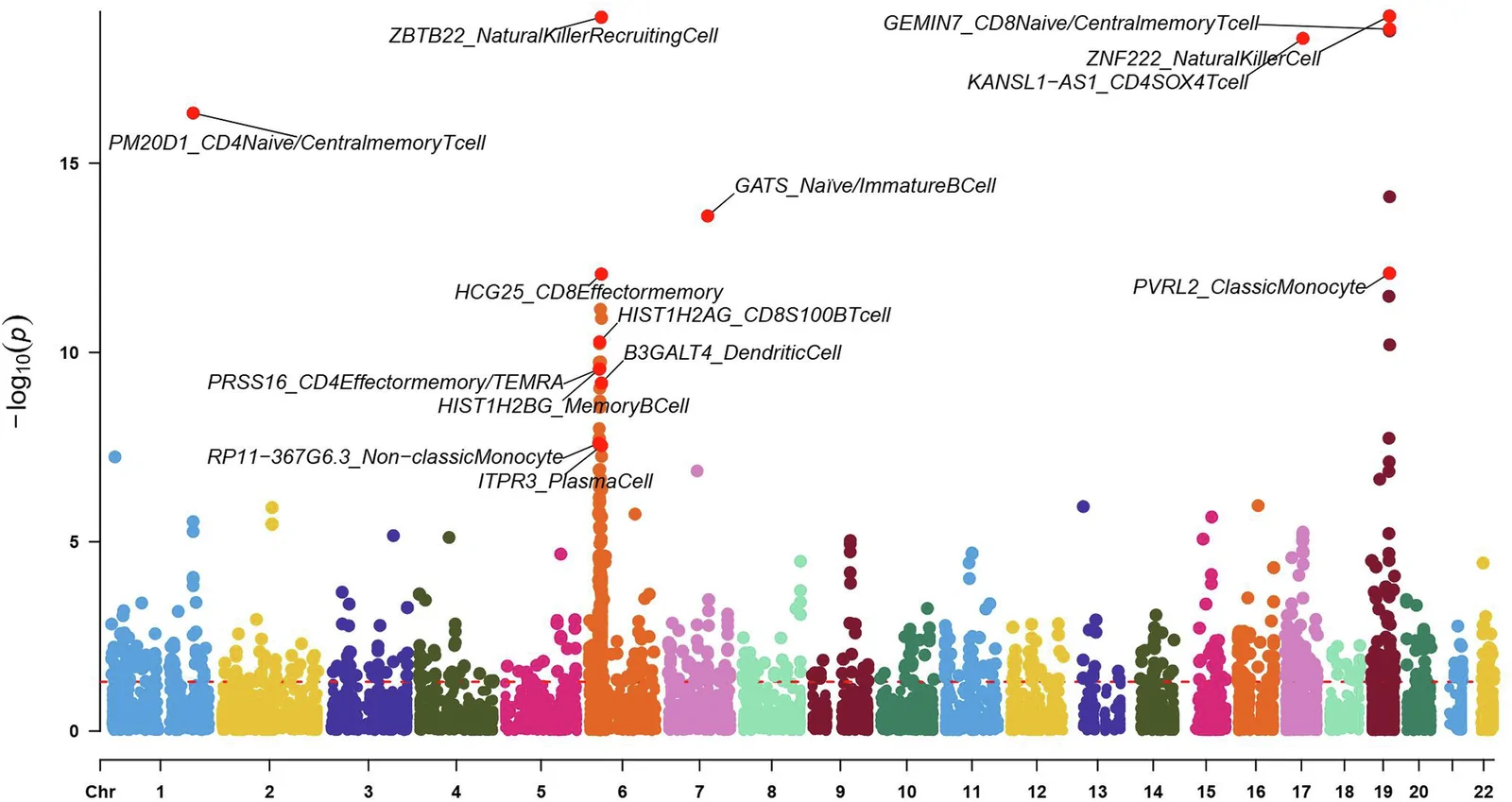
Causal Roles of Immune Cell-Targeted Gene Expression in AD. Each dot represents an eGene–cell type pair tested for association with AD risk. Red points indicate the most significant eGene associated with AD within each specific immune cell type. Notable eGenes showing strong cell-type-specific associations are annotated.

A Manhattan plot of cis-eQTL associations across the genome highlighted the most significant SNPs representing eGenes for each of the 14 immune cell types (Fig. 3). For each cell type, the SNP with the strongest association was selected as the representative variant for its corresponding eGene. Among these, PM20D1 showed the strongest signal in CD4 naïve/central memory T cells; PM20D1 encodes a peptidase involved in amide biosynthesis, amide catabolism, and amino acid metabolism and has been implicated in neurodegenerative processes. ZBTB22, the top hit in natural killer recruiting cells, is a transcription factor regulating gene transcription mediated by RNA polymerase II. GEMIN7, the most significant eGene in CD8 naïve/central memory T cells, encodes a core component of the SMN complex involved in snRNP assembly and pre-mRNA splicing, suggesting that regulatory variation in GEMIN7 may affect T-cell biology through RNA processing mechanisms. KANS1-AS1, prominent in CD4 SOX4⁺ T cells, is a long non-coding RNA with putative regulatory roles in transcription and chromatin-related processes, consistent with broader evidence linking lncRNAs to cellular stress responses and complex disease susceptibility. HIST1H2AG were the leading hits in CD8 S100B T cells, encoding a core histone protein critical for chromatin organization. B3GALT4 is a β-1,3-galactosyltransferase, significant in dendritic cells, which plays a key role in the biosynthesis of gangliosides. HIST1H2BG, the top gene in memory B cells, also encodes a histone protein, emphasizing the role of chromatin dynamics in adaptive immunity. Classical monocytes were represented by PVRL2, a cell-adhesion molecule involved in immune signaling, with genetic variants previously associated with multiple sclerosis. Finally, ITPR3 was the most significant gene in plasma cells, encoding a calcium channel critical for immune cell activation.

### Prioritization of AD-associated eGenes in immune cells via colocalization

**Fig. 4:**
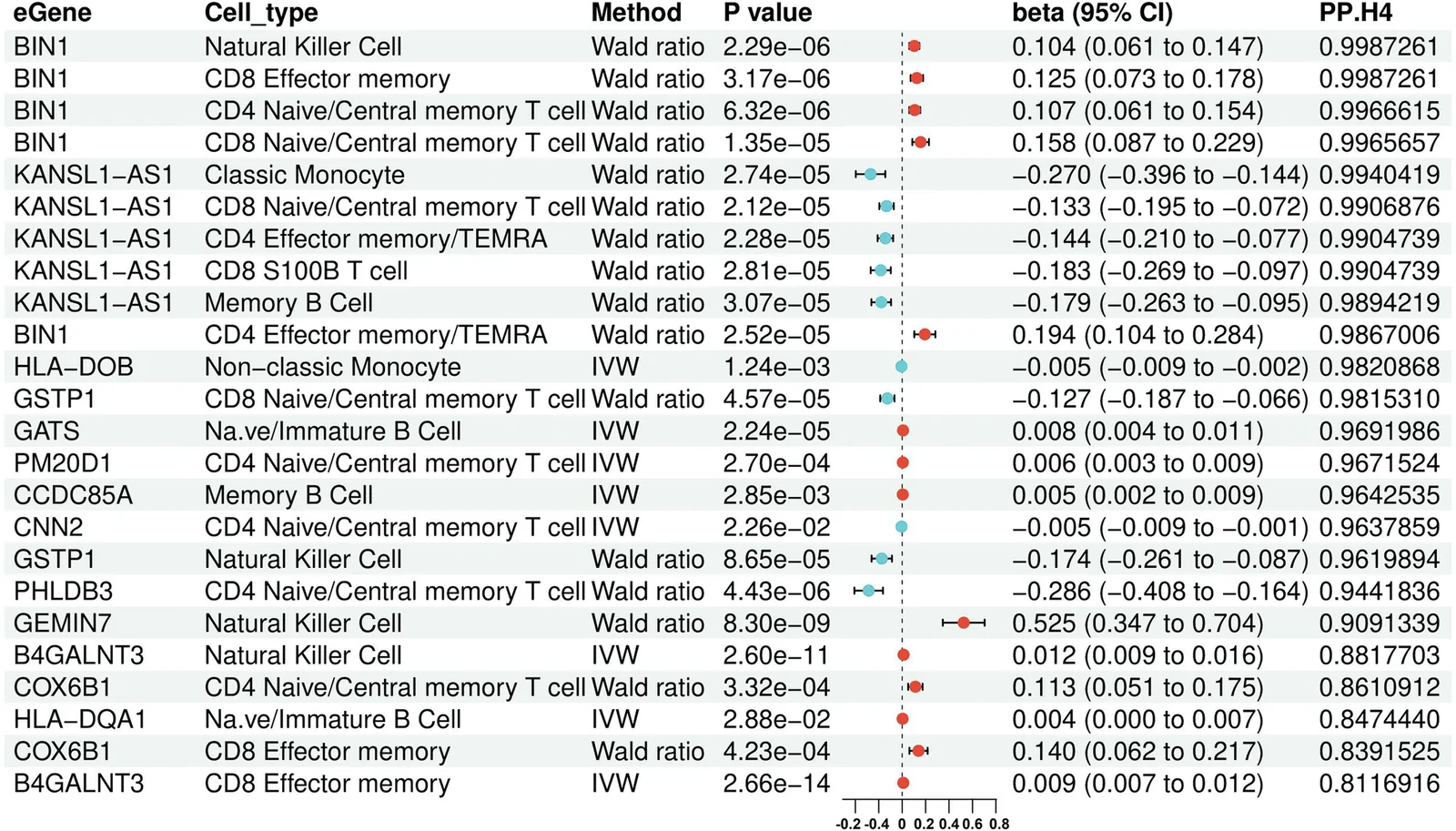
Prioritization of AD-Associated eGenes in Immune Cells via Colocalization. Each row represents an immune-cell-type–specific eGene, with points indicating beta estimates and horizontal bars denoting 95% confidence intervals. Effect estimates were derived using the Wald ratio or inverse-variance weighted (IVW) method. All associations shown meet the significance criteria (*P* < 0.05 and PP.H4 > 80%). Color indicates effect direction, with red markers representing positive beta values and blue markers representing negative beta values.

Through Bayesian colocalization, 24 immune cell–specific eGenes were prioritized, showing shared causal variants for gene expression and AD risk (Fig. 4, Tables S2–S3). Colocalization results were categorized using a two-level confidence scheme, defining strong support as PP.H4 > 0.80 and moderate support as 0.50 < PP.H4 ≤ 0.80. Only eGenes with strong colocalization evidence were carried forward for functional characterization and therapeutic prioritization, while those with moderate support were documented separately and interpreted conservatively. Within the high-confidence group, BIN1 demonstrated consistent colocalization signals across multiple immune cell populations, including natural killer cells, CD8 effector memory, CD4 naive/central memory, and CD8 naive/central memory T cells, with strong colocalization support and consistent risk effects. Other notable eGenes included KANSL1-AS1, HLA-DOB, and GSTP1, each displaying distinct patterns of cell-type specificity, as well as GATS, PM20D1, CCDC85A, CNN2, PHLDB3, GEMIN7, B4GALNT3, COX6B1, and HLA-DQA1 with progressively lower PP.H4 values but still above the strong-evidence threshold. Visualization via the chord diagram demonstrates that eGene–cell–AD associations include both common and cell-specific patterns (Table S1). Overall, the majority of prioritized eGenes were found in NK cells and CD4 NC T cells, highlighting their prominent contribution to the AD-related immune genetic architecture (Table 1).

**Table 1:** Prioritized eGenes Associated with AD Across Immune Cell Types.

Cell Type	eGene	nCount
**CD4 NC**	BIN1, PM20D1, CNN2, PHLDB3, COX6B1	5
**NK**	BIN1, GSTP1, GEMIN7, B4GALNT3	4
**CD8 ET**	BIN1, COX6B1, B4GALNT3	3
**CD8 NC**	BIN1, KANSL1-AS1, GSTP1	3
**CD4 ET**	BIN1, KANSL1-AS1	2
**B Mem**	KANSL1-AS1, CCDC85A	2
**B IN**	GATS, HLA-DQA1	2
**CD8 S100B**	KANSL1-AS1	1
**Mono C**	KANSL1-AS1	1
**Mono NC**	HLA-DOB	1

### Peripheral blood scRNA-seq validation of prioritized eGenes in AD

**Fig. 5:**
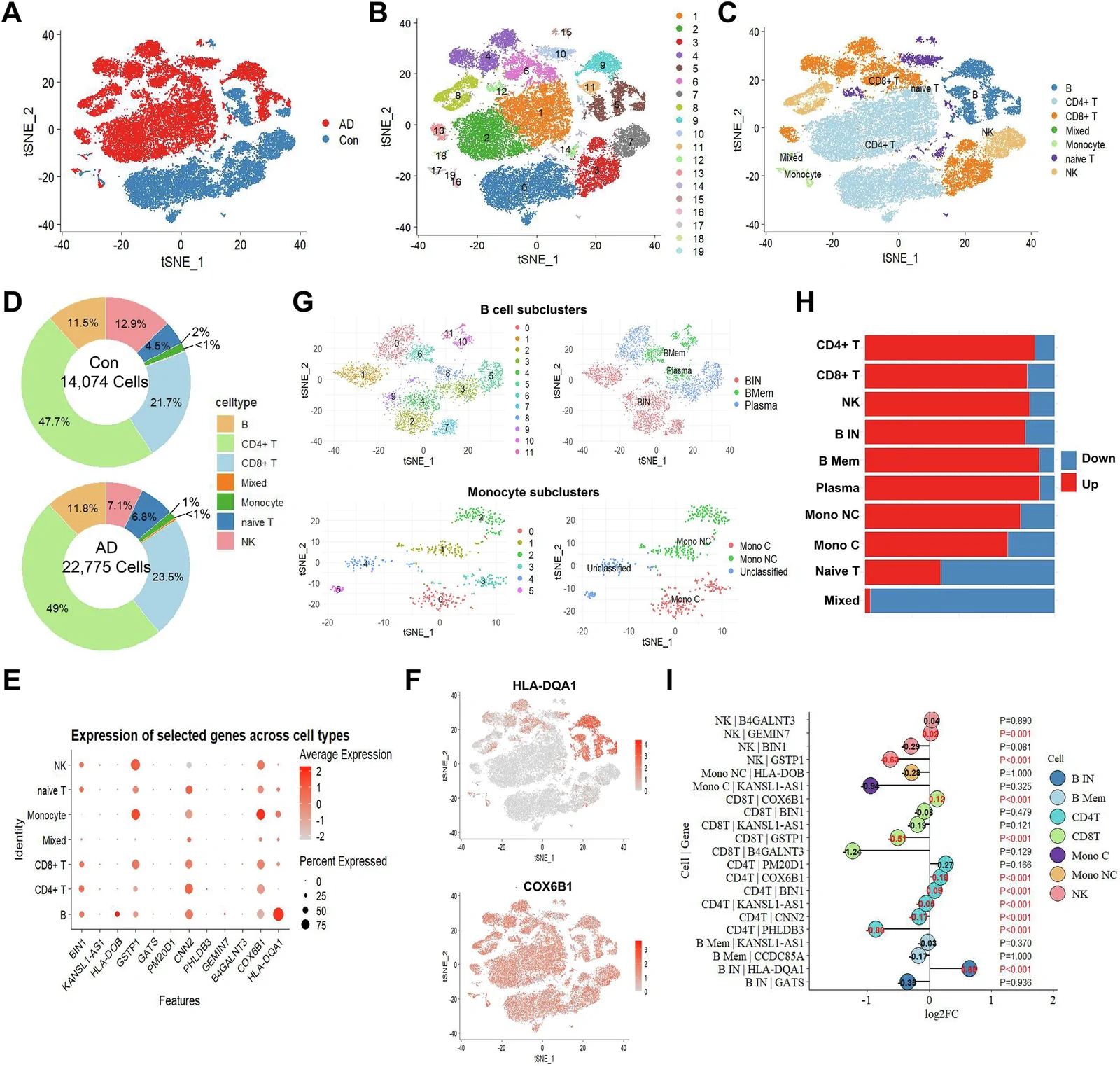
Peripheral Blood scRNA-seq Validation of Prioritized eGenes in AD. **A** tSNE projection of all cells from five samples, colored by sample identity. **B** tSNE projection colored by unsupervised clustering results. **C** Annotation of seven major immune cell types based on canonical marker genes. **D** Proportion of each major immune cell type in Con and AD groups. **E** Dot plot showing the expression levels and detection rates of genetically prioritized eGenes across major immune cell types. **F** Representative tSNE feature plots illustrating cell-type-specific expression patterns of HLA-DQA1 and COX6B1. **G** Subclusters of B cells and monocytes. **H** Proportion of significantly upregulated and downregulated genes per immune cell type. **I** Cell-type-specific differential expression of prioritized eGenes.

An independent peripheral blood scRNA-seq dataset (GSE181279) including two healthy controls and three AD patients was examined to explore the disease relevance of prioritized eGenes (Fig. 5A). After QC, cells were grouped into seven major immune cell types (Fig. 5B, C), with CD8⁺ and CD4⁺ T cells dominating in both AD and control samples (Fig. 5D). Several prioritized eGenes exhibited distinct, cell-type-specific expression patterns (Fig. 5E). Particularly, HLA-DQA1 showed high expression specifically in B cells, whereas COX6B1 showed widespread expression across multiple cell types (Fig. 5F). Subclustering further revealed heterogeneous expression patterns within B cells and monocytes (Fig. 5G). Although some discrepancies were noted between genetically predicted regulatory directions and observed transcriptional changes, differential expression analysis identified significant transcriptional changes associated with AD, including BIN1 upregulation in CD4⁺ T cells and HLA-DOB downregulation in non-classical monocytes, consistent with the direction of effects observed in the genetic analyses (Fig. 5H, I; Table S4).

### Phenome-wide screening of prioritized eGenes and IVs

**Fig. 6:**
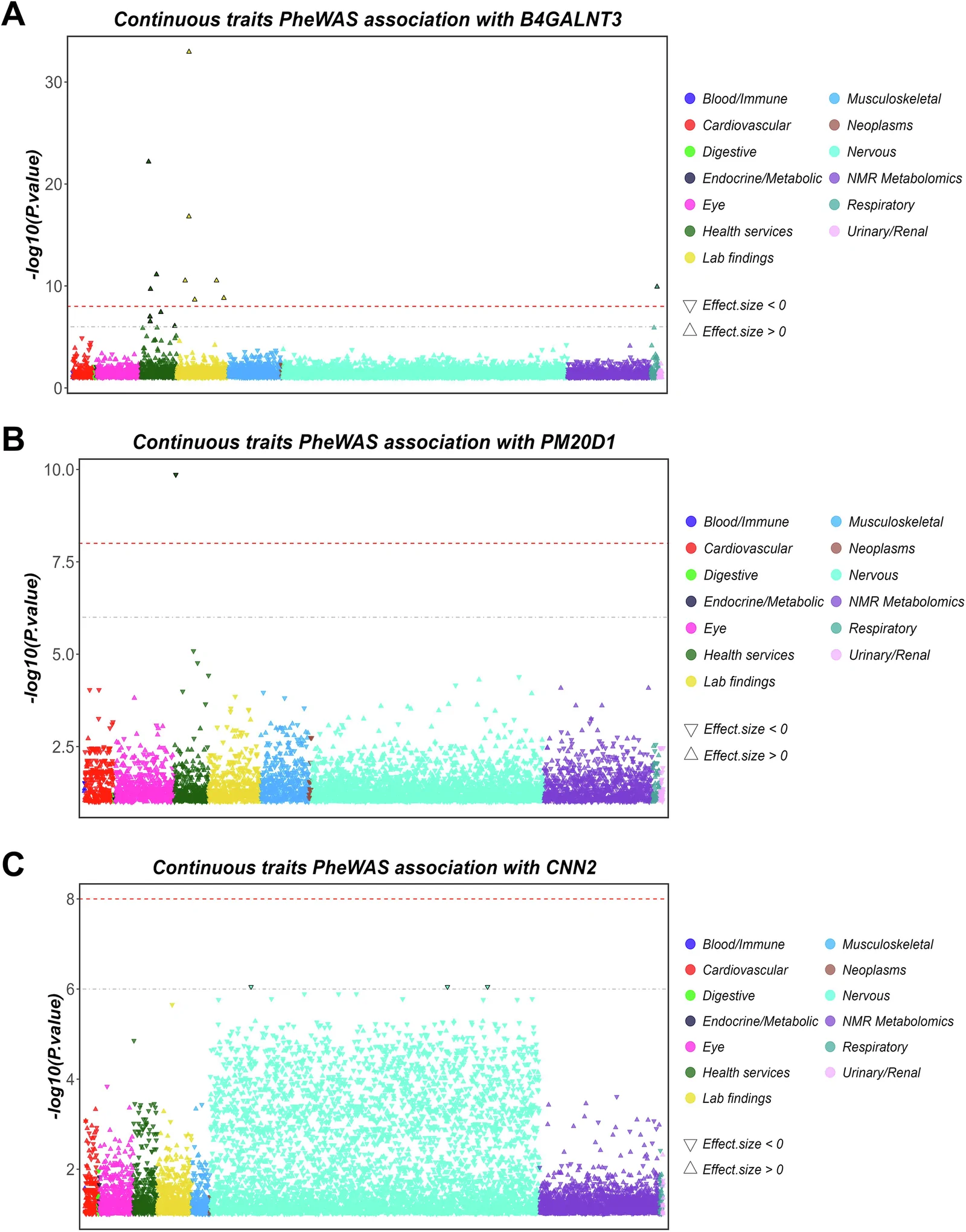
PheWAS analysis of prioritized eGenes by using AstraZeneca PheWAS Portal. **A** B4GALNT3. **B** PM20D1. **C** CNN2. Dot color indicates phenotype category; triangle direction reflects effect size direction.

**Fig. 7:**
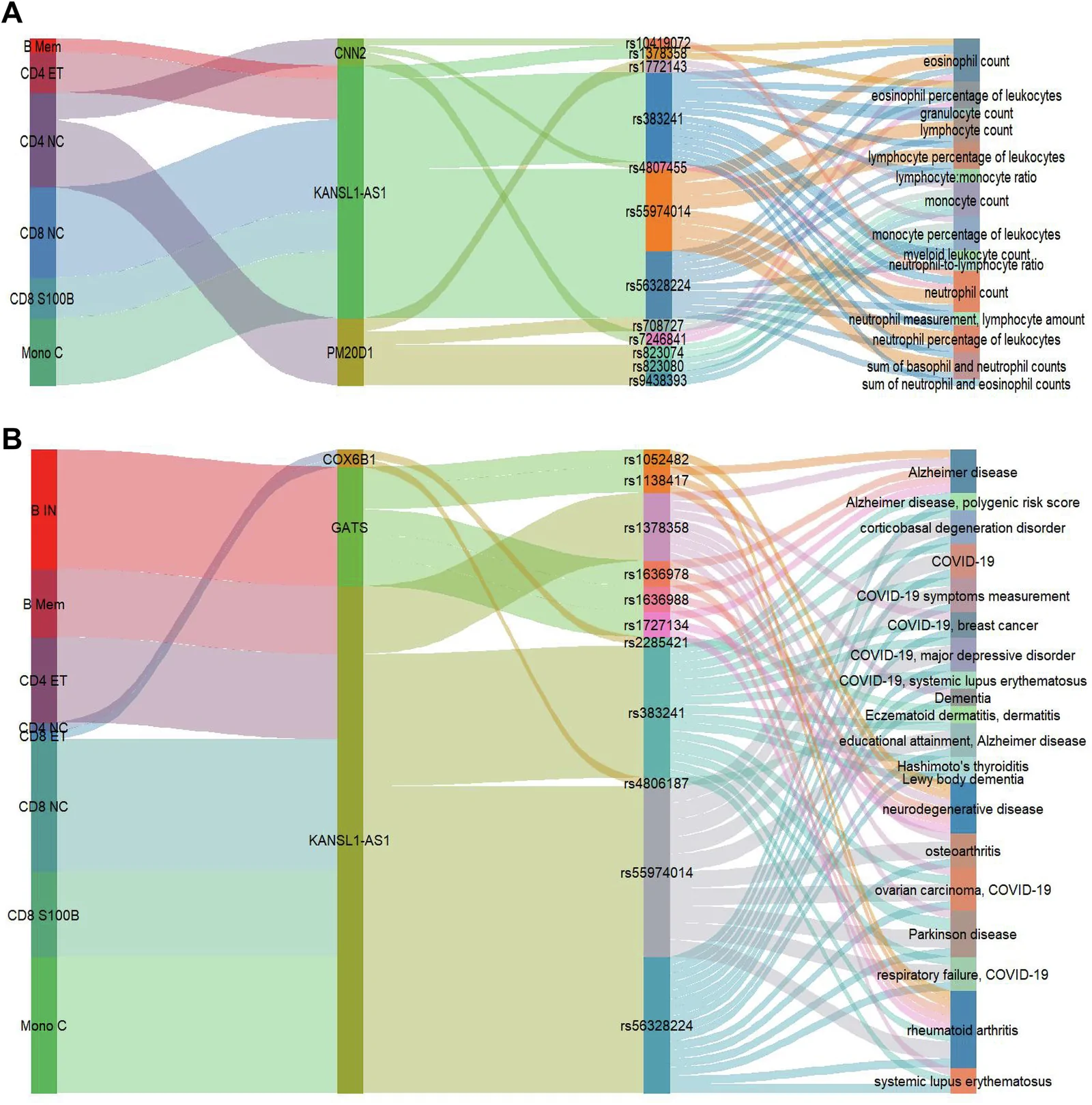
Immune/inflammation and neurodegenerative disease trait associations identified by SNP-level PheWAS in Open Targets Platform. Sankey plots illustrating significant immune/inflammation and neurodegenerative disease phenotypic associations for SNPs corresponding to prioritized eGenes. **A** Direct immune quantitative traits, including eosinophil count, eosinophil percentage of leukocytes, granulocyte count, lymphocyte count and percentage, lymphocyte-to-monocyte ratio, monocyte count and percentage, myeloid leukocyte count, neutrophil count, neutrophil percentage, neutrophil-to-lymphocyte ratio, neutrophil measurements, and combined basophil–neutrophil or neutrophil–eosinophil counts. **B** Immune/inflammation and neurodegenerative disease traits, including Alzheimer’s disease, Alzheimer’s disease polygenic risk score, corticobasal degeneration, dementia, Lewy body dementia, Parkinson’s disease, neurodegenerative disease, rheumatoid arthritis, systemic lupus erythematosus, Hashimoto’s thyroiditis, osteoarthritis, as well as COVID-19–related phenotypes and other inflammatory conditions. Cell types represent the source of the eGene, with links to the corresponding SNP and associated traits.

Phenome-wide association analyses at the gene and variant levels were conducted to evaluate the phenotypic diversity, immune associations, and potential safety profiles of leading targets. Using the AstraZeneca PheWAS Portal, we examined gene-level associations for eGenes with colocalization support. Most showed no genome-wide significant associations, consistent with a low risk of widespread off-target effects. Key exceptions comprised PM20D1, associated with factors influencing general health status and healthcare utilization; CNN2, linked to nervous system traits; and B4GALNT3, showing associations with health status, abnormal clinical findings, and respiratory system traits (Fig. 6; Tables S5–S7). At the SNP level, the instrumental variants employed in our MR analyses were examined via the Open Targets Platform. Several IVs demonstrated significant associations with immune-related traits, such as lymphocyte count and eosinophil count (Fig. 7A; Table S8), as well as with non-immune traits potentially relevant to immune/inflammation and neurodegenerative disease, including AD and Systemic lupus erythematosus (Fig. 7B; Table S9).

### Drug repurposing potential of targets specific to immune cell in AD

Through DSigDB/Enrichr, we identified compounds enriched for activity against prioritized AD eGenes, representing possible candidates for therapeutic repurposing (Table S10). Phenoxybenzamine, rimexolone, and NSC321521 ranked highly, exhibiting significant enrichment for GSTP1, BIN1, and HLA-DQA1, with combined scores above 200, suggesting effective targeting. GSTP1, in particular, was modulated by several compounds with varied clinical applications, including phenoxybenzamine (approved antihypertensive), meclizine (antiemetic), and aclarubicin (antineoplastic), whereas BIN1 was enriched for rimexolone, and HLA-DQA1 was associated with NSC321521, an immunomodulatory candidate. We next assessed the translational potential of these candidates by predicting their BBB permeability using the SwissADME platform (Table S12). Among the candidates, phenoxybenzamine and rimexolone were predicted to cross the BBB, suggesting potential central nervous system (CNS) activity, while NSC321521 is likely restricted to peripheral effects. This differentiation informs the pharmacokinetic considerations for prioritizing CNS-targeted versus peripheral immune-modulating therapies in AD.

### Molecular docking validation of drug-target interactions

**Fig. 8:**
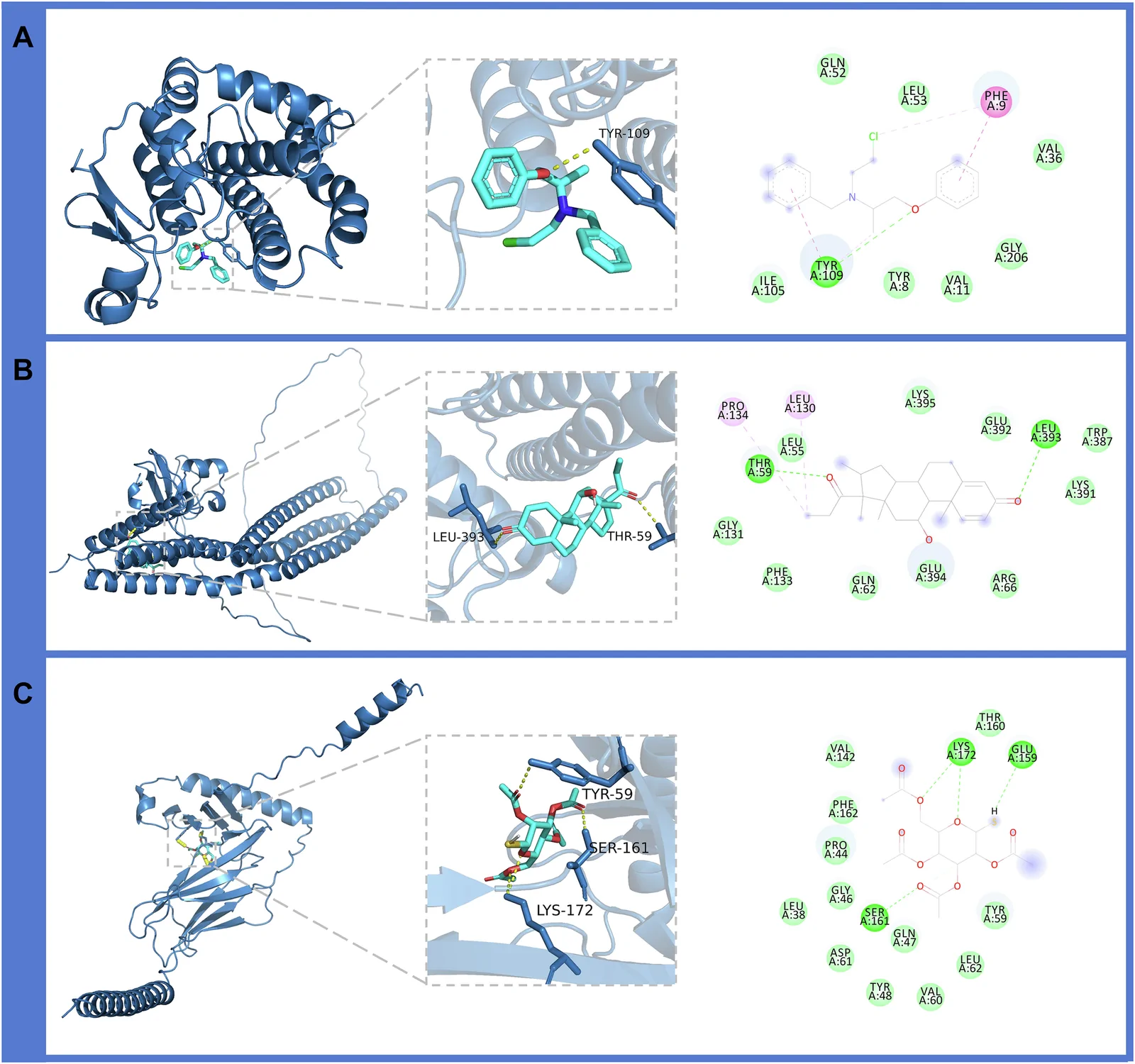
Molecular Docking Validation of Drug-Target Interactions. **A** phenoxybenzamine and GSTP1. **B** rimexolone and BIN1. **C** NSC321521 and HLA-DQA1. Left: protein-ligand complex; center: close-up of binding site; right: 2D interaction map highlighting key residues and binding types.

To evaluate the translational potential of the prioritized compounds, we performed molecular docking simulations focusing on top-ranked drug–target pairs identified from the DSigDB enrichment analysis (Fig. 8A–C; Table S12). Docking was restricted to candidate drugs with the highest combined scores and relevant eGene targets, incorporating BBB permeability predictions. Phenoxybenzamine, rimexolone, and NSC321521, which target GSTP1, BIN1, and HLA-DQA1, respectively, were prioritized for docking, and detailed molecular simulations were performed for all three compounds. Among these, BBB-permeant compounds such as phenoxybenzamine and rimexolone demonstrated favorable predicted binding energies to GSTP1 (−6.1 kcal/mol) and BIN1 (−7.6 kcal/mol), respectively, supporting potential central nervous system activity. In contrast, NSC321521, predicted as non–BBB-permeant, showed moderate binding to HLA-DQA1 (−5.6 kcal/mol), suggesting possible peripheral immune modulation. These results provide structural evidence that the top-ranked compounds are capable of engaging their respective targets and help prioritize candidates for further experimental validation in CNS- or periphery-focused AD interventions.

## Discussion

As a progressive neurodegenerative disease, AD imposes major clinical and societal burdens, with prevalence escalating in elderly populations [28]. Current therapeutic approaches remain largely symptomatic and fail to halt disease progression. Although GWAS have uncovered numerous AD-associated loci [29–31], leveraging this information to design cell-type-specific interventions remains a significant challenge. Immune dysregulation has emerged as a critical factor in AD pathogenesis [32–34], yet distinct immune cell types and underlying regulatory mechanisms remain incompletely understood. To address this gap, we integrated single-cell eQTL-based Mendelian randomization with Bayesian colocalization to systematically identify immune cell-specific genes causally linked to AD. A total of 13 candidate eGenes were prioritized, including BIN1, KANSL1-AS1, HLA-DOB, GSTP1, GATS, PM20D1, CCDC85A, CNN2, PHLDB3, GEMIN7, B4GALNT3, COX6B1, and HLA-DQA1, with HLA-DQA1, GSTP1, and BIN1 selected as key targets for drug repurposing and molecular docking validation. Peripheral blood single-cell RNA sequencing further confirmed cell-type-specific expression patterns consistent with genetic associations.

Although the sc-eQTL data used in this study were derived from peripheral blood immune cells, our findings should not be interpreted as implying that these cells directly substitute for central immune components such as microglia. Instead, we focus on genetically anchored regulatory variation in circulating immune cells that may influence AD susceptibility through systemic immune–CNS crosstalk, including cytokine signaling, blood–brain barrier integrity, and immune cell trafficking. By leveraging Mendelian randomization, the inferred effects reflect lifelong genetically driven differences in immune gene regulation that precede disease onset and are less susceptible to reverse causation.

BIN1, encoding the bridging integrator 1 protein, has been consistently identified as the second most significant genetic risk factor for late-onset Alzheimer’s disease (LOAD), following APOE [12, 27, 35, 36]. Its protein product regulates membrane curvature, endocytosis [37, 38], and vesicle trafficking [39], playing essential roles in synaptic vesicle recycling, endosomal-lysosomal homeostasis [40], neuronal calcium homeostasis and electrical activity [41]. MR and sc-eQTL analyses revealed strong colocalization of BIN1 in natural killer (NK) cells and CD4⁺/CD8⁺ T cell subsets, suggesting that genetically driven increases in BIN1 expression in these immune populations may contribute to AD risk. In T cells, BIN1 dysregulation could impair immune signaling, antigen presentation, and cytotoxic responses, potentially affecting microglial activation and amyloid-β clearance. In NK cells, altered BIN1 expression may influence cytotoxic activity and interactions with stressed neurons, thereby exacerbating neuroinflammatory cascades. Beyond its immune role, BIN1 has been shown to modulate Tau pathology in neurons, with isoform-specific reductions in AD patients linked to enhanced Tau aggregation, propagation, and impaired BDNF/TrkB endosomal signaling [37]. Conditional knockout or knockdown studies in mice neurons further demonstrate that BIN1 is critical for presynaptic neurotransmitter release, dendritic arborization, and neuronal electrical activity, implicating it in hippocampal degeneration and cognitive decline [39, 42]. Taken together, these findings position BIN1 at the intersection of immune dysregulation and canonical AD neuropathology, highlighting its dual impact on peripheral immune function and central neuronal vulnerability and underscoring its potential as a mechanistic and therapeutic target.

GSTP1 encodes Glutathione-S-transferase pi 1, a key regulator of cellular redox homeostasis and stress response. Consistent with its roles in vascular and immune cells, we found that GSTP1 is predominantly expressed in NK and CD8 NC T cells, where it participates in lysosomal function, protein degradation, and the maintenance of mitochondrial and metabolic homeostasis. Dysregulation of GSTP1 may lead to oxidative stress accumulation, impaired lysosomal clearance, and increased susceptibility to apoptosis in T cells, thereby promoting chronic low-grade inflammation. GSTP1 functions as a stress-responsive regulatory factor under chronic pathological conditions rather than a constitutive homeostatic enzyme. Accumulating evidence indicates that GSTP1 limits oxidative stress–driven cellular dysfunction through multiple converging mechanisms. In cardiovascular disease models, GSTP1 restrains angiotensin II–induced ferroptosis and tissue injury, thereby attenuating pathological remodeling [43]. In the immune system, GSTP1 acts downstream of DCAF1 to suppress excessive ROS accumulation and delay regulatory T cell senescence, preventing immune imbalance and chronic low-grade inflammation [44]. Moreover, GSTP1 modulates protein homeostasis by facilitating ubiquitin-mediated degradation of pathological signaling receptors independent of its catalytic transferase activity [45]. Collectively, these findings highlight GSTP1 as a key regulator of cellular stress adaptation, immune aging, and inflammation, processes that are highly relevant to neuroinflammatory environments and age-related neurodegenerative disorders such as AD, suggesting a potential strategy to mitigate both peripheral immune dysregulation and CNS-associated neuroinflammation in AD.

HLA-DQA1 is a classical MHC class II gene that is essential for shaping adaptive immune responses by facilitating peptide presentation within the CD4⁺ T cell axis [46]. Its genetically predicted expression was significantly associated with an increased risk of AD in B IN cells. This cell-type-specific association indicates distinct regulatory mechanisms through which B cell–mediated antigen presentation contributes to AD susceptibility. Notably, HLA class II variants have been repeatedly implicated in genome-wide association studies of autoimmune disorders such as systemic lupus erythematosus–associated pulmonary arterial hypertension [47] and multiple sclerosis [48], suggesting shared immunogenetic pathways linking aberrant humoral immunity and neurodegeneration. It is plausible that elevated HLA-DQA1 expression in B cells enhances antigen presentation and promotes sustained activation of adaptive immune responses, thereby facilitating chronic peripheral inflammation. Such immune dysregulation may indirectly amplify neuroinflammatory processes through cytokine release and immune–CNS crosstalk, ultimately contributing to neuronal dysfunction and disease progression in AD.

COX6B1, a subunit of cytochrome c oxidase (complex IV) in the mitochondrial electron transport chain, shows functional relevance in CD4⁺ and CD8⁺ T cell subsets. Genetic variants in COX6B1 are associated with increased risk of AD [49], likely by affecting mitochondrial function and cellular bioenergetics in these peripheral T cells. Notably, disease-associated COX6B1 mutations have also been linked to severe infantile encephalomyopathy [50], highlighting its critical role in neural and systemic cellular physiology.

KANSL1‑AS1, a long noncoding RNA, is associated with AD in B cells, T cells, and monocyte subsets. It participates in chromatin remodeling and transcriptional regulation, and its dysregulation in B cells may alter antigen presentation and adaptive immune responses, thereby modulating inflammatory processes that contribute to AD pathogenesis. This gene is located within a locus (Chr17:44 ± 1 Mb) previously implicated in PD [51, 52], AD [53] and lipid metabolism traits [54, 55], highlighting its potential role in immune-mediated mechanisms underlying disease progression. HLA-DOB, another non-classical monocyte-associated gene, encodes a non-classical MHC class II molecule that regulates antigen loading and presentation. Altered expression of HLA-DOB could shift non-classical monocyte-mediated immune responses, thereby impacting neuroinflammation and peripheral-CNS immune crosstalk. Notably, previous studies have demonstrated a negative association between HLA-DOB and rheumatoid arthritis [56], supporting its potential protective role in immune-mediated conditions.

The remaining candidate genes, including GATS, PM20D1, CCDC85A, CNN2, PHLDB3, GEMIN7, and B4GALNT3, exhibited more variable immune cell associations. Among them, PM20D1 is a metabolically active enzyme implicated in multiple neurodegenerative disorders. Beyond its epigenetically regulated neuroprotective role in AD [57], PM20D1 has been reported to possess enzymatic activity toward the generation of N-acyl amino acids [58], which directly inhibits α-synuclein aggregation and attenuates dopaminergic neurodegeneration in PD models [59], highlighting a conserved role in modulating protein aggregation and neuronal vulnerability through metabolic pathways. CNN2 plays a key role in lysosomal quality control by coordinating actin remodeling during lysophagy in response to lysosomal membrane damage. Its ubiquitylation and subsequent removal by VCP/p97, assisted by HSPB1, are required for efficient clearance of damaged lysosomes [60]. Given that lysosomal dysfunction is a hallmark of neurodegenerative diseases, CNN2 may indirectly influence neuronal proteostasis and vulnerability in conditions such as AD [61]. GEMIN7, like GEMIN5, is a component of the survival motor neuron complex involved in the assembly of small nuclear ribonucleoproteins (snRNPs) critical for pre-mRNA splicing [62]. Perturbation of GEMIN7 may similarly disrupt snRNP complex formation and RNA processing, potentially contributing to neuronal dysfunction and vulnerability in neurodegenerative contexts.

Drug repurposing analyses identified several small-molecule compounds with predicted interactions with prioritized AD-associated eGenes, exhibiting distinct pharmacokinetic properties. Among them, phenoxybenzamine and rimexolone were predicted to be BBB permeant, supporting their potential to engage CNS-relevant targets such as GSTP1 and BIN1, respectively. Overall, GSTP1 acts as a critical stress-sensing and protective hub, coordinating redox balance, protein quality control, and immune homeostasis to counteract chronic inflammation and neurodegenerative stress. Beyond this predicted interaction, Phenoxybenzamine may modulate AD-relevant pathways by inhibiting HDACs, and influencing oxidative stress, synaptic function, and neuroinflammation, with clinical evidence supporting its potential central activity [63]. rimexolone may act not only through systemic anti-inflammatory effects [64, 65] but also via direct engagement of BIN1-associated pathways. Such direct and indirect mechanisms may converge on endocytic trafficking, membrane dynamics, and tau pathology, collectively contributing to the restoration of neuronal homeostasis and attenuation of neurodegenerative progression in AD. In contrast, NSC321521, which was associated with HLA-DQA1, was predicted to have limited BBB permeability, suggesting a predominantly peripheral mode of action [66, 67]. Compounds with limited CNS penetration may exert indirect effects on AD progression by modulating peripheral immune mechanisms, including antigen presentation and immune activation mediated by B cells and T cells. Through peripheral-central immune crosstalk, such modulation could influence neuroinflammation and disease trajectory. Together, these findings highlight complementary therapeutic strategies targeting both central and peripheral immune-related pathways in AD, particularly relevant to early stages of disease progression or immune-mediated pathology.

A key question is whether these immune associations are independent or merely secondary to brain-driven genetic effects. While pleiotropic variants can influence multiple tissues, our use of Mendelian randomization (MR) provides quantitative evidence of a directed causal link from immune gene expression to AD risk. Furthermore, stringent Bayesian colocalization (PP.H4 > 0.80) specifically anchors AD-risk signals to gene expression within discrete immune populations. The identification of cell-type-specific targets—such as HLA-DQA1 in B cells and GSTP1 in NK cells—highlights regulatory landscapes distinct from canonical neuro-centric pathways. Although some loci like BIN1 may share regulatory architectures across the brain and periphery, this causal evidence, combined with colocalization, underscores that peripheral immune cells are genetically programmed components of AD risk, not merely passive markers of central pathology.

The focus on peripheral immune data was primarily driven by the availability of high-resolution sc-eQTL resources with sufficient power for robust causal inference, a depth currently lacking in brain-resident or CSF immune datasets. Since Mendelian randomization leverages genetic instruments representing lifelong systemic regulatory predispositions, these peripheral signals serve as an informative window into the baseline immune landscape that precedes neurodegeneration. While these findings do not substitute for the specialized roles of central microglia, they highlight genetically anchored regulatory axes that influence disease susceptibility through systemic-CNS crosstalk.

Furthermore, while our sc-eQTL data are derived from healthy individuals, we interpret these signals as representing lifelong, baseline regulatory predispositions that precede disease onset, rather than reactive transcriptional changes.

Several novel contributions are highlighted in this study. First, the use of sc-eQTLs across 14 immune cell types enabled high-resolution identification of cell-type-specific causal genes. Second, integrating scTWMR, Bayesian colocalization, and scRNA-seq validation established robust causal inference and functional relevance. Third, combining drug repurposing with molecular docking allowed prioritization of both CNS-penetrant and peripheral-targeting pharmacological candidates. Furthermore, the integrative framework developed in this study is inherently disease-agnostic and holds significant potential for generalization to other neurodegenerative conditions with known immune involvement. By replacing the AD GWAS summary statistics with those of Parkinson’s disease (PD), multiple sclerosis (MS), or amyotrophic lateral sclerosis (ALS), this pipeline could systematically prioritize cell-type-specific regulatory drivers across diverse pathological contexts. Given that systemic inflammation and peripheral immune dysregulation are emerging as shared hallmarks of neurodegeneration, applying this sc-eQTL-anchored approach to other disorders may reveal conserved or disease-specific immune-regulatory hubs, facilitating the discovery of broad-spectrum immunomodulatory therapies.

Collectively, this framework links genetic discovery with mechanistic and translational insights, facilitating precision immune-targeted interventions in AD.

Several limitations should be noted. Analyses were restricted to cohorts of European ancestry, potentially limiting generalizability. Furthermore, the validation scRNA-seq dataset is constrained by a small sample size (*n* = 5), which may not fully represent the broader transcriptomic landscape of AD. Consequently, these data were used strictly as a qualitative reference to provide supportive and illustrative evidence of cell-type-specific expression, rather than for independent quantitative discovery or robust statistical inference. We have interpreted these findings conservatively to avoid overinterpretation of this limited validation cohort. Similarly, the findings from our PheWAS are intended to provide supportive biological context rather than a conclusive safety validation of the prioritized targets. While PheWAS can highlight potential pleiotropic effects, these genetic associations do not substitute for rigorous pre-clinical toxicity or safety assessments. We have maintained this distinction and interpreted these findings conservatively to avoid overinterpretation of both the limited validation cohort and the genetic safety signals. Similarly, candidate compounds were prioritized using DSigDB, which integrates both direct biochemical targets (D1/D2) and expression-based signatures (D3/D4). To mitigate false positives inherent in expression-based data, by leveraging quantitative bioactivity thresholds from the database—we aimed to prioritize interactions with higher mechanistic relevance. Nonetheless, these pharmacological inferences remain hypothesis-generating and do not substitute for definitive biochemical assays. Experimental validation of predicted gene functions and drug-target interactions is lacking, and some immune subsets had limited statistical power due to small sample sizes. Predicted pharmacological effects remain hypothesis-generating until tested in functional or clinical studies. Future work should focus on cross-ancestry validation, functional assays, and preclinical evaluation to confirm mechanistic and therapeutic relevance. Despite these limitations, the prioritized cell-type-specific genes and candidate compounds provide a high-confidence roadmap for downstream experimental and translational studies in AD.

## Supplementary information

## Data Availability

All data used to support the findings of this study are included within the article, and raw data are available from the corresponding author.

## Code availability

This article does not have its own special program; the calculation scripts refers to https://github.com/DeepVasc-Lab/3-step_sceQTLMR.git [25].
