# Supplementary material for: Immune cell-specific genetic architecture of Alzheimer’s disease revealed by multi-omics analysis for therapeutic target discovery and prioritization
Source: Transl Psychiatry. 2026 Jun 23;16:449. doi: 10.1038/s41398-026-04199-9 (PMC13538538; doi:10.1038/s41398-026-04199-9)
Supplement: Supplementary file 1 [file 41398_2026_4199_MOESM10_ESM.pdf]

Table S10. All candidate drugs predicted to target prioritized AD-associated eGenes from DSigDB.

Term	*P*-value	Odds Ratio	Combined Score	Genes
phenoxybenzamine CTD 00006514	0.00712850018242009	166.475	822.9948695	GSTP1
meclizine CTD 00006252	0.00712850018242009	166.475	822.9948695	GSTP1
aclarubicin CTD 00007056	0.00712850018242009	166.475	822.9948695	GSTP1
PLATINUM CTD 00006576	0.007774217207607984	151.33333333333334	735.0172993	GSTP1
Oxibendazole CTD 00000598	0.007774217207607984	151.33333333333334	735.0172993	GSTP1
nitrendipine CTD 00006401	0.007774217207607984	151.33333333333334	735.0172993	GSTP1
equilin CTD 00005912	0.008419546757221652	138.71527777777777	662.6705253	GSTP1
benzyl isothiocyanate CTD 00001509	0.009064489027893918	128.03846153846155	602.2149226	GSTP1
ALLYL ISOTHIOCYANATE CTD 00000143	0.009064489027893918	128.03846153846155	602.2149226	GSTP1
Phylloquinone CTD 00006555	0.009064489027893918	128.03846153846155	602.2149226	GSTP1
dienestrol CTD 00005809	0.009709044215672634	118.88690476190476	551.0048325	GSTP1
CHLOROBENZENE CTD 00001495	0.010353212518883667	110.95555555555555	507.117753	GSTP1
terbinafine CTD 00001890	0.010353212518883667	110.95555555555555	507.117753	GSTP1
doxepin CTD 00005873	0.010996994142530848	104.015625	469.1243344	GSTP1
tranilast CTD 00000646	0.010996994142530848	104.015625	469.1243344	GSTP1
estradiol valerate CTD 00000816	0.010996994142530848	104.015625	469.1243344	GSTP1
N-Hydroxy phip CTD 00002510	0.011640389286531055	97.8921568627451	435.9406355	GSTP1
1,8,9-Trihydroxyanthracene CTD 00005413	0.012283398155032895	92.44907407407408	406.7303182	GSTP1
ERIODICTYOL CTD 00000357	0.012283398155032895	92.44907407407408	406.7303182	GSTP1
doxycycline CTD 00005875	0.012283398155032895	92.44907407407408	406.7303182	GSTP1
Chloroethylene CTD 00006592	0.012283398155032895	92.44907407407408	406.7303182	GSTP1
sulfinpyrazone CTD 00006807	0.01292602095289387	87.57894736842105	380.8381799	GSTP1
phenolphthalein CTD 00007346	0.013568257886165577	83.19583333333334	357.7439297	GSTP1
VERLUKAST CTD 00002469	0.013568257886165577	83.19583333333334	357.7439297	GSTP1
hypericin CTD 00000183	0.013568257886165577	83.19583333333334	357.7439297	GSTP1
84625-61-6 CTD 00007215	0.013568257886165577	83.19583333333334	357.7439297	GSTP1
Styrene oxide CTD 00000717	0.013568257886165577	83.19583333333334	357.7439297	GSTP1
artemisinin CTD 00001499	0.013568257886165577	83.19583333333334	357.7439297	GSTP1
merbromin CTD 00006270	0.013568257886165577	83.19583333333334	357.7439297	GSTP1
Oltipraz CTD 00001231	0.014210109163131231	79.23015873015873	337.0293803	GSTP1
CAPTAN CTD 00005571	0.014210109163131231	79.23015873015873	337.0293803	GSTP1
ifosfamide CTD 00006138	0.014851574986337721	75.625	318.3547328	GSTP1
rimexolone HL60 UP	0.014851574986337721	75.625	318.3547328	BIN1

Term	*P*-value	Odds Ratio	Combined Score	Genes
CUMENE HYDROPEROXIDE CTD 00000338	0.014851574986337721	75.625	318.3547328	GSTP1
fenbendazole CTD 00005958	0.01549265556616628	72.33333333333333	301.4411523	GSTP1
teniposide CTD 00006838	0.01549265556616628	72.33333333333333	301.4411523	GSTP1
Crystal violet CTD 00006023	0.016133351108423025	69.31597222222223	286.0577742	GSTP1
mebendazole CTD 00006249	0.016133351108423025	69.31597222222223	286.0577742	GSTP1
iodoquinol CTD 00005833	0.01741358791764657	63.9775641025641	259.1414089	GSTP1
Galangin CTD 00001740	0.01741358791764657	63.9775641025641	259.1414089	GSTP1
pyrimethamine CTD 00006670	0.01741358791764657	63.9775641025641	259.1414089	GSTP1
phenylbutazone CTD 00006518	0.01741358791764657	63.9775641025641	259.1414089	GSTP1
NSC321521 CTD 00005452	0.01805312960024282	61.60493827160494	247.3090958	HLA-DQA1
thiotepa CTD 00006869	0.01805312960024282	61.60493827160494	247.3090958	GSTP1
Pararosaniline CTD 00000224	0.018692287080739334	59.401785714285715	236.3979777	GSTP1
beta-Lapachone CTD 00000762	0.018692287080739334	59.401785714285715	236.3979777	GSTP1
ETHACRYNIC ACID	0.01933106056430268	57.35057471264368	226.3077835	GSTP1
Microcystin LR CTD 00002422	0.019969450260865367	55.43611111111111	216.9520847	GSTP1
Plumbagin CTD 00000768	0.019969450260865367	55.43611111111111	216.9520847	GSTP1
2,4-Diisocyanato-1-methylbenzene CTD 000	0.019969450260865367	55.43611111111111	216.9520847	GSTP1
