# Supplementary material for: Immune cell-specific genetic architecture of Alzheimer’s disease revealed by multi-omics analysis for therapeutic target discovery and prioritization
Source: Transl Psychiatry. 2026 Jun 23;16:449. doi: 10.1038/s41398-026-04199-9 (PMC13538538; doi:10.1038/s41398-026-04199-9)
Supplement: Supplementary file 2 [file 41398_2026_4199_MOESM11_ESM.pdf]

Table S11. Corresponding SMILES of the top 3 predicted compounds.

Term	SMILES
phenoxybenzamine CTD 00006514	CC(COC1=CC=CC=C1)N(CCCl)CC2=CC=CC=C2
rimexolone HL60 UP	CCC(=O)[C@]1([C@@H](C[C@@H]2[C@@]1(C[C@@H]([C@H]3[C@H]2CCC4=CC(=O)C=C[C@]34C)O)C)C)C
NSC321521 CTD 00005452	CC[PH+](CC)CC.CC(=O)OCC1C(C(C(C(O1)[S-])OC(=O)C)OC(=O)C)OC(=O)C.[Au+]
