# Supplementary material for: Immune cell-specific genetic architecture of Alzheimer’s disease revealed by multi-omics analysis for therapeutic target discovery and prioritization
Source: Transl Psychiatry. 2026 Jun 23;16:449. doi: 10.1038/s41398-026-04199-9 (PMC13538538; doi:10.1038/s41398-026-04199-9)
Supplement: Supplementary file 3 [file 41398_2026_4199_MOESM12_ESM.pdf]

Table S12. Detailed information on molecular docking simulation.

Drug	Target	BBB permeant	UniProt ID	Binding energy (kcal/mol)
phenoxybenzamine	GSTP1	√	P28801	-6.1
rimexolone	BIN1	√	O00499-10	-7.6
NSC321521	HLA-DQA1	×	P01909	-5.6
