# Supplementary material for: Immune cell-specific genetic architecture of Alzheimer’s disease revealed by multi-omics analysis for therapeutic target discovery and prioritization
Source: Transl Psychiatry. 2026 Jun 23;16:449. doi: 10.1038/s41398-026-04199-9 (PMC13538538; doi:10.1038/s41398-026-04199-9)
Supplement: Supplementary file 4 [file 41398_2026_4199_MOESM1_ESM.pdf]

Table S1. Detailed information on instruments used in MR analysis.

Cell_type	Cell_ID	Ensembl_ID	eGene	SNP	Effect Allele	Other Allele	Beta	SE	EAF	P-value	Chr
Natural Killer Cell	nk	ENSG00000136717	BIN1	rs10194375	A	C	0.606535452	0.028614934	0.338358	1.0294E-99	2
CD8 Effector memory	cd8et	ENSG00000136717	BIN1	rs10194375	A	C	0.50189907	0.02976776	0.338358	8.78548E-64	2
CD4 Naive/Central memory T cell	cd4nc	ENSG00000136717	BIN1	rs11682128	A	G	0.563784894	0.029126337	0.343332	1.79572E-83	2
CD8 Naive/Central memory T cell	cd8nc	ENSG00000136717	BIN1	rs11682128	A	G	0.383346036	0.030723302	0.343332	9.92088E-36	2
Classic Monocyte	monoc	ENSG00000214401	KANSL1-AS1	rs56328224	T	C	0.393976324	0.030856103	0.206545	2.46854E-37	17
CD8 Naive/Central memory T cell	cd8nc	ENSG00000214401	KANSL1-AS1	rs383241	A	G	0.769086549	0.025971249	0.205728	1.0165E-192	17
CD8 Naive/Central memory T cell	cd8nc	ENSG00000214401	KANSL1-AS1	rs560699746	A	C	0.159414244	0.031747437	0.397598	5.13159E-07	17
CD4 Effector memory/TEMRA	cd4et	ENSG00000214401	KANSL1-AS1	rs55974014	A	C	0.722085084	0.026876565	0.199353	5.3801E-159	17
CD8 S100B T cell	cd8s100b	ENSG00000214401	KANSL1-AS1	rs55974014	A	C	0.565739582	0.029119112	0.199353	4.43447E-84	17
Memory B Cell	bmem	ENSG00000214401	KANSL1-AS1	rs1378358	T	C	0.562202324	0.029144136	0.190299	6.46592E-83	17
CD4 Effector memory/TEMRA	cd4et	ENSG00000136717	BIN1	rs11682128	A	G	0.31216276	0.031150158	0.343332	1.22968E-23	2
Non-classic Monocyte	mononc	ENSG00000241106	HLA-DOB	rs13212435	T	C	-0.327755773	0.031855586	0.154034	7.91561E-25	6
Non-classic Monocyte	mononc	ENSG00000241106	HLA-DOB	rs204999	G	A	0.268753609	0.032160213	0.274068	6.4491E-17	6
Non-classic Monocyte	mononc	ENSG00000241106	HLA-DOB	rs9268560	G	C	-0.238289435	0.032291776	0.576101	1.59171E-13	6
Non-classic Monocyte	mononc	ENSG00000241106	HLA-DOB	rs204992	A	G	0.23220561	0.032316017	0.201817	6.69788E-13	6
Non-classic Monocyte	mononc	ENSG00000241106	HLA-DOB	rs6415128	A	G	-0.219486015	0.032364544	0.633	1.18786E-11	6
CD8 Naive/Central memory T cell	cd8nc	ENSG00000084207	GSTP1	rs614080	A	G	0.428445986	0.030398222	0.495119	4.1088E-45	11
Natural Killer Recruiting Cell	nkr	ENSG00000214401	KANSL1-AS1	rs56328224	T	C	0.320787448	0.031312257	0.206545	1.24898E-24	17
Natural Killer Recruiting Cell	nkr	ENSG00000214401	KANSL1-AS1	rs34186148	C	G	-0.144776134	0.031996539	0.37065	6.04688E-06	17
Naïve/Immature B Cell	bin	ENSG00000160844	GATS	rs1052482	T	A	-0.200730503	0.031626226	0.707607	2.19606E-10	7
Naïve/Immature B Cell	bin	ENSG00000160844	GATS	rs1727134	C	T	0.31210444	0.031150467	0.726024	1.25446E-23	7
Naïve/Immature B Cell	bin	ENSG00000160844	GATS	rs1138417	G	C	0.311684903	0.031152686	0.724299	1.44785E-23	7
Naïve/Immature B Cell	bin	ENSG00000160844	GATS	rs1636978	A	G	0.312301928	0.031149421	0.726039	1.1725E-23	7
Naïve/Immature B Cell	bin	ENSG00000160844	GATS	rs1636988	A	G	0.312075772	0.031150618	0.72373	1.26682E-23	7
CD4 Naive/Central memory T cell	cd4nc	ENSG00000162877	PM20D1	rs1772143	A	T	-0.188240447	0.031665921	0.395827	2.77175E-09	1
CD4 Naive/Central memory T cell	cd4nc	ENSG00000162877	PM20D1	rs9438393	G	A	0.319770113	0.03110932	0.396489	8.77077E-25	1
CD4 Naive/Central memory T cell	cd4nc	ENSG00000162877	PM20D1	rs823080	A	G	0.320254126	0.031106684	0.396181	7.3952E-25	1
CD4 Naive/Central memory T cell	cd4nc	ENSG00000162877	PM20D1	rs708727	A	G	0.319061326	0.031113172	0.396951	1.12535E-24	1
CD4 Naive/Central memory T cell	cd4nc	ENSG00000162877	PM20D1	rs823074	C	T	0.314611438	0.031137134	0.397028	5.3002E-24	1
Memory B Cell	bmem	ENSG00000055813	CCDC85A	rs6714635	T	C	0.190256409	0.031659695	0.567893	1.86188E-09	2
Memory B Cell	bmem	ENSG00000055813	CCDC85A	rs11125626	G	T	-0.390528065	0.030674486	0.576856	3.95859E-37	2
Memory B Cell	bmem	ENSG00000055813	CCDC85A	rs62164393	T	A	-0.38540563	0.030709415	0.572867	3.97106E-36	2
Memory B Cell	bmem	ENSG00000055813	CCDC85A	rs17047856	C	A	-0.383554242	0.030721903	0.575193	9.04651E-36	2
Memory B Cell	bmem	ENSG00000055813	CCDC85A	rs13021782	G	A	-0.383422413	0.030722789	0.574623	9.5908E-36	2
CD4 Naive/Central memory T cell	cd4nc	ENSG00000064666	CNN2	rs4807455	A	G	0.371256119	0.030803029	0.255559	1.87962E-33	19
CD4 Naive/Central memory T cell	cd4nc	ENSG00000064666	CNN2	rs7246841	C	T	-0.246040973	0.031459548	0.712042	5.24603E-15	19
CD4 Naive/Central memory T cell	cd4nc	ENSG00000064666	CNN2	rs10419072	A	G	-0.155523493	0.031757371	0.133123	9.71917E-07	19
Natural Killer Cell	nk	ENSG00000084207	GSTP1	rs656652	T	C	0.311834231	0.031151896	0.49518	1.37585E-23	11
CD4 Naive/Central memory T cell	cd4nc	ENSG00000176531	PHLDB3	rs35681564	A	G	0.292492058	0.031250653	0.186341	8.00767E-21	19
Natural Killer Cell	nk	ENSG00000142252	GEMIN7	rs6509193	G	A	-0.236978388	0.031495766	0.639144	5.30706E-14	19
Natural Killer Cell	nk	ENSG00000139044	B4GALNT3	rs7314691	A	G	0.431024481	0.030378272	0.260025	1.07823E-45	12
Natural Killer Cell	nk	ENSG00000139044	B4GALNT3	rs1056008	C	T	-0.271861497	0.031348289	0.262997	4.2351E-18	12

Cell_type	Cell_ID	Ensembl_ID	eGene	SNP	Effect Allele	Other Allele	Beta	SE	EAF	P-value	Chr
Natural Killer Cell	nk	ENSG00000139044	B4GALNT3	rs7298766	G	A	-0.260928877	0.031396865	0.295581	9.5166E-17	12
Natural Killer Cell	nk	ENSG00000139044	B4GALNT3	rs11063529	A	G	-0.226949423	0.031534156	0.218047	6.15789E-13	12
Natural Killer Cell	nk	ENSG00000139044	B4GALNT3	rs11063536	C	T	-0.243698322	0.03146905	0.218063	9.62888E-15	12
CD4 Naive/Central memory T cell	cd4nc	ENSG00000126267	COX6B1	rs4806187	G	A	-0.41700102	0.030484954	0.516307	1.35707E-42	19
Naïve/Immature B Cell	bin	ENSG00000196735	HLA-DQA1	rs5002178	A	G	0.356571054	0.030895808	0.61797	8.18887E-31	6
Naïve/Immature B Cell	bin	ENSG00000196735	HLA-DQA1	rs9268848	A	G	-0.145224737	0.031782447	0.498753	4.89268E-06	6
CD8 Effector memory	cd8et	ENSG00000126267	COX6B1	rs2285421	C	T	-0.337401961	0.03101036	0.507977	1.43094E-27	19
CD8 Effector memory	cd8et	ENSG00000139044	B4GALNT3	rs7314691	A	G	0.357780206	0.030888335	0.260025	5.02442E-31	12
CD8 Effector memory	cd8et	ENSG00000139044	B4GALNT3	rs11063534	C	G	-0.183947957	0.031678949	0.213166	6.37426E-09	12
CD8 Effector memory	cd8et	ENSG00000139044	B4GALNT3	rs11063529	A	G	-0.158878119	0.031748821	0.218047	5.60884E-07	12
CD8 Effector memory	cd8et	ENSG00000139044	B4GALNT3	rs11063536	C	T	-0.156137404	0.03175582	0.218063	8.79649E-07	12
CD8 Effector memory	cd8et	ENSG00000139044	B4GALNT3	rs11063537	G	A	-0.156137404	0.03175582	0.218094	8.79649E-07	12
