# Supplementary material for: Immune cell-specific genetic architecture of Alzheimer’s disease revealed by multi-omics analysis for therapeutic target discovery and prioritization
Source: Transl Psychiatry. 2026 Jun 23;16:449. doi: 10.1038/s41398-026-04199-9 (PMC13538538; doi:10.1038/s41398-026-04199-9)
Supplement: Supplementary file 5 [file 41398_2026_4199_MOESM2_ESM.pdf]

Table S2. Summary of eGenes associated with AD in MR analysis.

eGene	Cell_type	Method	beta	se	Pval	or	PP.H4
BIN1	Natural Killer Cell	Wald ratio	0.103768377870737	0.0219556212657028	2.28672E-06	1.10934347665766	0.998726123
BIN1	CD8 Effector memory	Wald ratio	0.125402105285758	0.0269130928292176	3.16947E-06	1.13360418967026	0.998726122
BIN1	CD4 Naive/Central memory T cell	Wald ratio	0.107193365161904	0.0237392973170134	6.31855E-06	1.11314947799933	0.996661512
BIN1	CD8 Naive/Central memory T cell	Wald ratio	0.157648688990011	0.0362248566379629	1.34933E-05	1.17075482339281	0.996565672
KANSL1-AS1	Classic Monocyte	Wald ratio	-0.269856317	0.0643486663458505	2.74491E-05	0.763489186703906	0.994041878
KANSL1-AS1	CD8 Naive/Central memory T cell	Wald ratio	-0.133445319	0.0313815550391399	2.1154E-05	0.875075317492166	0.990687555
KANSL1-AS1	CD4 Effector memory/TEMRA	Wald ratio	-0.143539871	0.0338870461851509	2.27706E-05	0.866286259634458	0.990473948
KANSL1-AS1	CD8 S100B T cell	Wald ratio	-0.183207969	0.0437395880904427	2.80671E-05	0.832594983395539	0.990473947
KANSL1-AS1	Memory B Cell	Wald ratio	-0.178791506	0.0428957052137275	3.07215E-05	0.836280240779962	0.989421855
BIN1	CD4 Effector memory/TEMRA	Wald ratio	0.193597724438343	0.0459501212574916	2.51762E-05	1.21360797991488	0.986700615
HLA-DOB	Non-classic Monocyte	IVW	-0.00534841	0.00165633309161119	0.001241948	0.994665866954894	0.982086818
GSTP1	CD8 Naive/Central memory T cell	Wald ratio	-0.126513264	0.0310324781892923	4.56594E-05	0.881162461938217	0.981531047
GATS	Naïve/Immature B Cell	IVW	0.00753771410754059	0.00177806561432317	2.2424E-05	1.00756619418779	0.969198618
PM20D1	CD4 Naive/Central memory T cell	IVW	0.00566095805320752	0.00155394364927856	0.000269518	1.00567701155468	0.967152402
CCDC85A	Memory B Cell	IVW	0.00517017572316806	0.00173308704518782	0.002852352	1.00518356414523	0.96425348
CNN2	CD4 Naive/Central memory T cell	IVW	-0.00503854	0.00220915153903265	0.022562763	0.994974131797879	0.963785929
GSTP1	Natural Killer Cell	Wald ratio	-0.174124245	0.0443556033411649	8.64985E-05	0.840192501371517	0.96198944
PHLDB3	CD4 Naive/Central memory T cell	Wald ratio	-0.285971525	0.0623023535186365	4.43119E-06	0.751284008228782	0.944183602
GEMIN7	Natural Killer Cell	Wald ratio	0.525347484061869	0.0911711128960946	8.30175E-09	1.69104635795534	0.909133875
B4GALNT3	Natural Killer Cell	IVW	0.01213587257076	0.00182009115347362	2.59794E-11	1.0122098110723	0.881770305
COX6B1	CD4 Naive/Central memory T cell	Wald ratio	0.112940970641354	0.0314685849312878	0.00033194	1.11956584374429	0.861091188
HLA-DQA1	Naïve/Immature B Cell	IVW	0.00376457153245412	0.0017222002605875	0.028822328	1.00377166643216	0.847443978
COX6B1	CD8 Effector memory	Wald ratio	0.139624262600618	0.0396095132600265	0.000423453	1.14984167915832	0.839152465
B4GALNT3	CD8 Effector memory	IVW	0.00943177871680505	0.00123877228574153	2.66129E-14	1.00947639811145	0.811691564
