# Supplementary material for: Immune cell-specific genetic architecture of Alzheimer’s disease revealed by multi-omics analysis for therapeutic target discovery and prioritization
Source: Transl Psychiatry. 2026 Jun 23;16:449. doi: 10.1038/s41398-026-04199-9 (PMC13538538; doi:10.1038/s41398-026-04199-9)
Supplement: Supplementary file 6 [file 41398_2026_4199_MOESM3_ESM.pdf]

Table S3. Colocalization results for eGenes associated with AD.

CELL_TYPE	GENE	H0	H1	H2	H3	H4
Natural Killer Cell	BIN1	7.1577557458165e-16	0.00127387671654966	5.61171858670242e-16	0	0.998726123
CD8 Effector memory	BIN1	6.88219507499173e-10	0.00127387671498561	5.39567755468827e-10	0	0.998726122
CD4 Naive/Central memory T cell	BIN1	7.24816015076304e-13	0.00333848750294674	2.16384283370756e-13	0	0.996661512
CD8 Naive/Central memory T cell	BIN1	7.40534110962259e-05	0.00333816647037768	2.21076714061429e-05	0	0.996565672
Classic Monocyte	KANSL1-AS1	0.000770066216380434	0.00503605649032225	0.00015199949985214	0	0.994041878
CD8 Naive/Central memory T cell	KANSL1-AS1	8.30971504365617e-25	0.00931244539779847	8.84013910888232e-26	0	0.990687555
CD4 Effector memory/TEMRA	KANSL1-AS1	1.04876715117034e-19	0.00952605152988953	1.09045866273778e-20	0	0.990473948
CD8 S100B T cell	KANSL1-AS1	1.08146935743493e-09	0.00952605151851619	1.12446087578579e-10	0	0.990473947
Memory B Cell	KANSL1-AS1	1.85037446698869e-09	0.0105781428484544	1.73073947292849e-10	0	0.989421855
CD4 Effector memory/TEMRA	BIN1	0.00769655721707613	0.00330512178050794	0.00229770587735112	0	0.986700615
Non-classic Monocyte	HLA-DOB	0.0106712761806575	0.00472935900031505	0.00242147180923544	9.10753141001688e-05	0.982086818
CD8 Naive/Central memory T cell	GSTP1	2.44364548272068e-06	0.0184663799641828	1.29885441141898e-07	0	0.981531047
Naïve/Immature B Cell	GATS	0.0115940555247557	0.00899908910946552	0.0062929589347332	0.00391527843966567	0.969198618
CD4 Naive/Central memory T cell	PM20D1	0.00529095335566897	0.0221711907676624	0.00122391539821274	0.0041615380155276	0.967152402
Memory B Cell	CCDC85A	5.23098268498122e-05	0.0324455352572875	6.78129732475092e-06	0.00324189352306835	0.96425348
CD4 Naive/Central memory T cell	CNN2	0.0112420788759954	0.0245251734908201	0.000443369994576876	3.44852614828899e-06	0.963785929
Natural Killer Cell	GSTP1	0.0194851474532093	0.0174513090648228	0.00107410315315664	0	0.96198944
CD4 Naive/Central memory T cell	PHLDB3	0.00642764045641285	0.000123186896062917	0.0492655705523189	0	0.944183602
Natural Killer Cell	GEMIN7	5.82251073835706e-14	5.8255392548445e-16	0.0908661245093516	0	0.909133875
Natural Killer Cell	B4GALNT3	0.000192961877823433	0.0935489889263829	5.222154110496e-05	0.024435522549065	0.881770305
CD4 Naive/Central memory T cell	COX6B1	5.40162578306342e-05	0.138854461123988	3.34976083813611e-07	0	0.861091188
Naïve/Immature B Cell	HLA-DQA1	0.0108043493766121	0.140236402959219	0.000169011197474277	0.00134625798055352	0.847443978
CD8 Effector memory	COX6B1	0.0284699657735823	0.13219684915919	0.000180720207064975	0	0.839152465
CD8 Effector memory	B4GALNT3	0.04637932304614	0.0848960389648096	0.0204364401770283	0.0365966341215503	0.811691564
