# Supplementary material for: Immune cell-specific genetic architecture of Alzheimer’s disease revealed by multi-omics analysis for therapeutic target discovery and prioritization
Source: Transl Psychiatry. 2026 Jun 23;16:449. doi: 10.1038/s41398-026-04199-9 (PMC13538538; doi:10.1038/s41398-026-04199-9)
Supplement: Supplementary file 7 [file 41398_2026_4199_MOESM4_ESM.pdf]

Table S4. Differential gene expression summary across diversr cell types in AD.

Cell type	Direction	nGenes	Percentage
CD4+ T	Down	358	10.46%
	Up	3064	89.54%
CD8+ T	Down	451	14.39%
	Up	2683	85.61%
NK	Down	366	13.10%
	Up	2428	86.90%
naive T	Down	1388	59.91%
	Up	929	40.09%
B IN	Down	355	15.40%
	Up	1955	84.60%
B Mem	Down	225	8.06%
	Up	2565	91.90%
Plasma	Down	197	7.80%
	Up	2329	92.20%
Mono NC	Down	273	18%
	Up	1247	82%
Mono C	Down	374	24.70%
	Up	1140	75.30%
Mixed	Down	1435	97.16%
	Up	42	2.84%
