# Supplementary material for: Immune cell-specific genetic architecture of Alzheimer’s disease revealed by multi-omics analysis for therapeutic target discovery and prioritization
Source: Transl Psychiatry. 2026 Jun 23;16:449. doi: 10.1038/s41398-026-04199-9 (PMC13538538; doi:10.1038/s41398-026-04199-9)
Supplement: Supplementary file 8 [file 41398_2026_4199_MOESM5_ESM.pdf]

Table S5. PheWAS analysis of PM20D1 by using AstraZeneca PheWAS Portal.

eGene	Class	Phenotypic category	P-value	Effect size	Collapsing.model
PM20D1	Continuous	Chapter XXI Factors influencing health status and contact with health services	1.38852852021012E-10	-4.586511354	rec
