# Supplementary material for: Immune cell-specific genetic architecture of Alzheimer’s disease revealed by multi-omics analysis for therapeutic target discovery and prioritization
Source: Transl Psychiatry. 2026 Jun 23;16:449. doi: 10.1038/s41398-026-04199-9 (PMC13538538; doi:10.1038/s41398-026-04199-9)
Supplement: Supplementary file 9 [file 41398_2026_4199_MOESM6_ESM.pdf]

Table S6. PheWAS analysis of CNN2 by using AstraZeneca PheWAS Portal.

eGene	Class	Phenotypic category	P-value	Effect size	Collapsing.model
CNN2	Continuous	Chapter VI Diseases of the nervous system	8.93016E-07	-4.586511354	ptvraredmg
CNN2	Continuous	Chapter VI Diseases of the nervous system	8.93016E-07	-4.586511354	raredmg
CNN2	Continuous	Chapter VI Diseases of the nervous system	8.93016E-07	-4.586511354	flexdmg
