# Supplementary material for: Immune cell-specific genetic architecture of Alzheimer’s disease revealed by multi-omics analysis for therapeutic target discovery and prioritization
Source: Transl Psychiatry. 2026 Jun 23;16:449. doi: 10.1038/s41398-026-04199-9 (PMC13538538; doi:10.1038/s41398-026-04199-9)
Supplement: Supplementary file 10 [file 41398_2026_4199_MOESM7_ESM.pdf]

Table S7. PheWAS analysis of B4GALNT3 by using AstraZeneca PheWAS Portal.

eGene	Class	Phenotypic category	P-value	Effect size	Collapsing.model
B4GALNT3	Continuous	Chapter XXI Factors influencing health status and contact with health services	6.50058E-23	0.111649153	flexdmg
B4GALNT3	Continuous	Chapter XXI Factors influencing health status and contact with health services	1.01387E-07	0.137386661	ptv5pcnt
B4GALNT3	Continuous	Chapter XXI Factors influencing health status and contact with health services	1.01387E-07	0.137386661	ptv
B4GALNT3	Continuous	Chapter XXI Factors influencing health status and contact with health services	3.02144E-07	0.05894573	flexnonsynmtr
B4GALNT3	Continuous	Chapter XXI Factors influencing health status and contact with health services	2.00875E-10	0.079681258	flexdmg
B4GALNT3	Continuous	Chapter XXI Factors influencing health status and contact with health services	7.47274E-12	0.11052123	ptvraredmg
B4GALNT3	Continuous	Chapter XXI Factors influencing health status and contact with health services	3.70769E-08	0.066763327	flexdmg
B4GALNT3	Continuous	Chapter XXI Factors influencing health status and contact with health services	8.45077E-07	0.049404521	flexdmg
B4GALNT3	Continuous	Chapter XVIII Symptoms signs and abnormal clinical and laboratory findings not elsewhere classified	2.98679E-11	0.245152781	ptv5pcnt
B4GALNT3	Continuous	Chapter XVIII Symptoms signs and abnormal clinical and laboratory findings not elsewhere classified	1.55709E-17	0.197037691	ptvraredmg
B4GALNT3	Continuous	Chapter XVIII Symptoms signs and abnormal clinical and laboratory findings not elsewhere classified	1.09615E-33	0.196137382	flexdmg
B4GALNT3	Continuous	Chapter XVIII Symptoms signs and abnormal clinical and laboratory findings not elsewhere classified	2.23775E-09	0.177308231	raredmg
B4GALNT3	Continuous	Chapter XVIII Symptoms signs and abnormal clinical and laboratory findings not elsewhere classified	2.98679E-11	0.245152781	ptv
B4GALNT3	Continuous	Chapter XVIII Symptoms signs and abnormal clinical and laboratory findings not elsewhere classified	1.5366E-09	0.099491293	flexnonsynmtr
B4GALNT3	Continuous	Chapter X Diseases of the respiratory system	1.20371E-10	0.056769762	flexdmg
