# Supplementary material for: Immune cell-specific genetic architecture of Alzheimer’s disease revealed by multi-omics analysis for therapeutic target discovery and prioritization
Source: Transl Psychiatry. 2026 Jun 23;16:449. doi: 10.1038/s41398-026-04199-9 (PMC13538538; doi:10.1038/s41398-026-04199-9)
Supplement: Supplementary file 11 [file 41398_2026_4199_MOESM8_ESM.pdf]

Table S8. Significant immune-related trait associations of IVs used in MR analyses from Open Targets Platform.

Cell type	Gene	SNP	LeadVariant	Trait	Phenotype	Study	P-value	Beta	PosteriorProbability	FineMappingMethod	FineMappingConfidence	TopL2G	l2GScore	CredibleSetSize
CD8 S100B	KANSL1-AS1	rs55974014	17_45591487_A_G	neutrophil (fraction, minimum, inv-norm transformed)	neutrophil count	GCST90475541	8.09169864654541x10-24	0.014084584748667382	0.0002005395485717409	SuSiE-inf	SuSiE fine-mapped credible set with out-of-sample LD	CRHR1	0.5099560618400574	2395
CD8 S100B	KANSL1-AS1	rs55974014	17_45618793_A_T	Sum basophil neutrophil counts	sum of basophil and neutrophil counts	GCST004620	2.3425774574279785x10-11	0.016323855245078723	0.0003559971444392398	SuSiE-inf	SuSiE fine-mapped credible set with out-of-sample LD	MAPT	0.25818219780921936	1733
CD8 S100B	KANSL1-AS1	rs55974014	17_45618793_A_T	Neutrophil count	neutrophil count	GCST004629	2.937805652618408x10-11	0.015872561504500494	0.00033554318676241396	SuSiE-inf	SuSiE fine-mapped credible set with out-of-sample LD	MAPT	0.17867916822433472	1809
CD8 S100B	KANSL1-AS1	rs55974014	17_45768114_T_C	lymphocyte (absolute count, minimum, inv-norm transformed)	lymphocyte count	GCST90475429	2.378000020980835x10-10	0.02187	0.0002474908955587464	PICS	PICS fine-mapped credible set extracted from summary statistics	MAPT	0.32434797286987305	2811
CD8 S100B	KANSL1-AS1	rs55974014	17_45768114_T_C	Lymphocyte count	lymphocyte count	GCST90002316	2.9720447063446045x10-14	0.008773098251850348	0.0005535750550092118	SuSiE-inf	SuSiE fine-mapped credible set with out-of-sample LD	MAPT	0.2875947654247284	1514
CD8 S100B	KANSL1-AS1	rs55974014	17_45768114_T_C	lymphocyte (absolute count, minimum, inv-norm transformed)	lymphocyte count	GCST90479665	6.980000019073486x10-9	0.01876	0.00025599920822647385	PICS	PICS fine-mapped credible set extracted from summary statistics	MAPT	0.34357011318206787	2881
CD8 S100B	KANSL1-AS1	rs55974014	17_45770203_TTTTGTTTG_T	Lymphocyte count	lymphocyte count	GCST90002388	3.5953187942504883x10-11	-0.00763271	0.0006012525322572461	SuSiE-inf	SuSiE fine-mapped credible set with out-of-sample LD	MAPT	0.21399348974227905	2506
CD8 S100B	KANSL1-AS1	rs55974014	17_45941153_T_C	lymphocyte (fraction, maximum, inv-norm transformed)	lymphocyte count	GCST90475432	4.342451095581055x10-15	-0.013841055	0.00047302120090526216	SuSiE-inf	SuSiE fine-mapped credible set with out-of-sample LD	MAPT	0.3556195795536041	2061
CD8 S100B	KANSL1-AS1	rs55974014	17_45720994_A_G	Eosinophil counts	eosinophil count	GCST90056180	4x10-14	NA	0.0002265119037909917	PICS	PICS fine-mapped credible set based on reported top hit	MAPT	0.1859586089849472	2682
CD8 S100B	KANSL1-AS1	rs55974014	17_46112492_G_A	Lymphocyte percentage of white cells	lymphocyte percentage of leukocytes	GCST004632	5.456538200378418x10-13	-0.017725684	0.00014278736202507608	SuSiE-inf	SuSiE fine-mapped credible set with out-of-sample LD	STH	0.3389870524406433	1707
CD8 S100B	KANSL1-AS1	rs55974014	17_46138530_C_T	Neutrophil percentage of white cells	neutrophil percentage of leukocytes	GCST004633	9.970807075500488x10-15	0.018819767160248347	0.000269327591756285	SuSiE-inf	SuSiE fine-mapped credible set with out-of-sample LD	STH	0.4624694883823395	1535
CD8 S100B	KANSL1-AS1	rs55974014	17_45948522_C_A	Lymphocyte percentage (UKB data field 30180)	lymphocyte percentage of leukocytes	GCST90309876	3.114000082015991x10-13	0.0295712	0.00021298239072317518	PICS	PICS fine-mapped credible set extracted from summary statistics	MAPT	0.41726210713386536	2724
B Mem	KANSL1-AS1	rs1378358	17_46720553_C_T	Eosinophill count (UKB data field 30150)	eosinophil count	GCST90013985	5.551758766174316x10-34	-0.018413858	0.06499785597893502	SuSiE-inf	SuSiE fine-mapped credible set with out-of-sample LD	ARL17A	0.3340194821357727	17
B Mem	KANSL1-AS1	rs1378358	17_46720553_C_T	Eosinophill percentage (UKB data field 30210)	eosinophil percentage of leukocytes	GCST90309879	1.1829999685287476x10-20	-0.0387456	0.003439918640854624	PICS	PICS fine-mapped credible set extracted from summary statistics	STH	0.12728427350521088	2308
B Mem	KANSL1-AS1	rs1378358	17_46709946_C_T	Eosinophil counts	eosinophil count	GCST90002381	3.372039794921875x10-35	-0.018490479	0.09627389600732486	SuSiE-inf	SuSiE fine-mapped credible set with out-of-sample LD	WNT3	0.29814428091049194	16
Mono C	KANSL1-AS1	rs56328224	17_45386127_A_C	neutrophil (absolute count, mean, inv-norm transformed)	neutrophil count	GCST90475529	9.381999969482422x10-10	0.02334	0.0001959930998205832	PICS	PICS fine-mapped credible set extracted from summary statistics	ARHGAP27	0.26441600918769836	2808
Mono C	KANSL1-AS1	rs56328224	17_45443309_T_C	Neutrophill count (UKB data field 30140)	neutrophil count	GCST90013984	7.261449337005615x10-19	0.010323402122700998	0.0003897758195415892	SuSiE-inf	SuSiE fine-mapped credible set with out-of-sample LD	ARHGAP27	0.33282899856567383	2186
Mono C	KANSL1-AS1	rs56328224	17_45578810_C_A	Eosinophil counts	eosinophil count	GCST90002381	3.2913196086883545x10-38	-0.020678356	0.0004052923126561498	SuSiE-inf	SuSiE fine-mapped credible set with out-of-sample LD	MAPT	0.288733571767807	2234
Mono C	KANSL1-AS1	rs56328224	17_45447800_C_CT	Neutrophil count	neutrophil count	GCST90002398	6.910816192626953x10-19	0.010611617230070865	0.0007980729707850372	SuSiE-inf	SuSiE fine-mapped credible set with out-of-sample LD	SPPL2C	0.14334917068481445	2494
Mono C	KANSL1-AS1	rs56328224	17_45591487_A_G	neutrophil (fraction, minimum, inv-norm transformed)	neutrophil count	GCST90475541	8.09169864654541x10-24	0.014084584748667382	0.0011321013199103046	SuSiE-inf	SuSiE fine-mapped credible set with out-of-sample LD	CRHR1	0.5099560618400574	2395
Mono C	KANSL1-AS1	rs56328224	17_45591486_C_T	lymphocyte (fraction, minimum, inv-norm transformed)	lymphocyte count	GCST90475438	5.3282389640808105x10-13	-0.01288517	0.0006194408441706503	SuSiE-inf	SuSiE fine-mapped credible set with out-of-sample LD	MAPT	0.3223322033882141	2303
Mono C	KANSL1-AS1	rs56328224	17_45618793_A_T	Sum basophil neutrophil counts	sum of basophil and neutrophil counts	GCST004620	2.3425774574279785x10-11	0.016323855245078723	0.0002504427735243258	SuSiE-inf	SuSiE fine-mapped credible set with out-of-sample LD	MAPT	0.25818219780921936	1733
Mono C	KANSL1-AS1	rs56328224	17_45618793_A_T	Neutrophil count	neutrophil count	GCST004629	2.937805652618408x10-11	0.015872561504500494	0.0002601783864438832	SuSiE-inf	SuSiE fine-mapped credible set with out-of-sample LD	MAPT	0.17867916822433472	1809
Mono C	KANSL1-AS1	rs56328224	17_45768114_T_C	lymphocyte (absolute count, minimum, inv-norm transformed)	lymphocyte count	GCST90475429	2.378000020980835x10-10	0.02187	0.00028935536615252837	PICS	PICS fine-mapped credible set extracted from summary statistics	MAPT	0.32434797286987305	2811
Mono C	KANSL1-AS1	rs56328224	17_45768114_T_C	lymphocyte (fraction, mean, inv-norm transformed)	lymphocyte count	GCST90475435	4.9070000648498535x10-18	0.02716	0.0002495074114313449	PICS	PICS fine-mapped credible set extracted from summary statistics	MAPT	0.2943761944770813	2612
Mono C	KANSL1-AS1	rs56328224	17_45768114_T_C	lymphocyte (absolute count, minimum, inv-norm transformed)	lymphocyte count	GCST90479665	6.980000019073486x10-9	0.01876	0.0002930498493856434	PICS	PICS fine-mapped credible set extracted from summary statistics	MAPT	0.34357011318206787	2881
Mono C	KANSL1-AS1	rs56328224	17_45770203_TTTTGTTTG_T	Lymphocyte count	lymphocyte count	GCST90002388	3.5953187942504883x10-11	-0.00763271	0.00033584195260956027	SuSiE-inf	SuSiE fine-mapped credible set with out-of-sample LD	MAPT	0.21399348974227905	2506
Mono C	KANSL1-AS1	rs56328224	17_45849924_G_A	Eosinophil counts	eosinophil count	GCST90134621	1x10-17	-0.038	0.00023799886923124282	PICS	PICS fine-mapped credible set based on reported top hit	SPPL2C	0.3401679992675781	2616
Mono C	KANSL1-AS1	rs56328224	17_45941153_T_C	lymphocyte (fraction, maximum, inv-norm transformed)	lymphocyte count	GCST90475432	4.342451095581055x10-15	-0.013841055	0.0016093914597107664	SuSiE-inf	SuSiE fine-mapped credible set with out-of-sample LD	MAPT	0.3556195795536041	2061
Mono C	KANSL1-AS1	rs56328224	17_45847931_C_T	neutrophil (fraction, maximum, inv-norm transformed)	neutrophil count	GCST90479713	7.110000133514404x10-18	0.02941	0.0002567103559630955	PICS	PICS fine-mapped credible set extracted from summary statistics	SPPL2C	0.3140776753425598	2621
Mono C	KANSL1-AS1	rs56328224	17_45948522_C_A	Neutrophill percentage (UKB data field 30200)	neutrophil percentage of leukocytes	GCST90309878	6.493000030517578x10-15	-0.0318963	0.0002473882112423339	PICS	PICS fine-mapped credible set extracted from summary statistics	MAPT	0.41726210713386536	2685
Mono C	KANSL1-AS1	rs56328224	17_45949182_T_C	eosinophil (absolute count, minimum, inv-norm transformed)	eosinophil count	GCST90479603	6.639999866485596x10-16	0.02772	0.000224850430671144	PICS	PICS fine-mapped credible set extracted from summary statistics	MAPT	0.4052606523036957	2627
Mono C	KANSL1-AS1	rs56328224	17_45720994_A_G	Eosinophil counts	eosinophil count	GCST90056180	4x10-14	NA	0.0002685761407031787	PICS	PICS fine-mapped credible set based on reported top hit	MAPT	0.1859586089849472	2682
Mono C	KANSL1-AS1	rs56328224	17_45745406_T_C	Eosinophil percentage of white cells	eosinophil percentage of leukocytes	GCST90002382	8.442001342773438x10-49	-0.022914564	0.002622385655488973	SuSiE-inf	SuSiE fine-mapped credible set with out-of-sample LD	CRHR1	0.30252769589424133	851
Mono C	KANSL1-AS1	rs56328224	17_46036724_CT_C	eosinophil (fraction, minimum, inv-norm transformed)	eosinophil count	GCST90475303	3.4830000400543213x10-28	-0.03702	0.00024247423348905641	PICS	PICS fine-mapped credible set extracted from summary statistics	MAPT	0.2499091923236847	2593
Mono C	KANSL1-AS1	rs56328224	17_46036724_CT_C	eosinophil (fraction, mean, inv-norm transformed)	eosinophil count	GCST90475300	6.54855489730835x10-49	-0.023639791	0.00019358215162618636	SuSiE-inf	SuSiE fine-mapped credible set with out-of-sample LD	MAPT	0.2379968911409378	2090
Mono C	KANSL1-AS1	rs56328224	17_46042134_C_CTT	Neutrophils and lymphocytes in blood (confirmatory factor anal	neutrophil measurement, lymphocyte amount	GCST90309369	2.139305591583252x10-23	0.01648682789443355	0.0004006177313778149	SuSiE-inf	SuSiE fine-mapped credible set with out-of-sample LD	MAPT	0.23639625310897827	2271
Mono C	KANSL1-AS1	rs56328224	17_46211866_G_T	Eosinophill count (UKB data field 30150)	eosinophil count	GCST90013985	2.6277098655700684x10-36	-0.019559268	0.0007643444600246766	SuSiE-inf	SuSiE fine-mapped credible set with out-of-sample LD	MAPT	0.23589292168617249	2258
Mono C	KANSL1-AS1	rs56328224	17_46236156_G_T	Lymphocyte-to-monocyte ratio	lymphocyte:monocyte ratio	GCST90056181	1x10-19	NA	0.00022830551601572737	PICS	PICS fine-mapped credible set based on reported top hit	STH	0.28819653391838074	2579
Mono C	KANSL1-AS1	rs56328224	17_46246819_A_G	Lymphocyte percentage of white cells	lymphocyte percentage of leukocytes	GCST90002389	1.1399171352386475x10-27	-0.016283486	0.0008524161048190427	SuSiE-inf	SuSiE fine-mapped credible set with out-of-sample LD	STH	0.27214905619621277	2404
Mono C	KANSL1-AS1	rs56328224	17_46255427_G_A	Neutrophil-to-lymphocyte ratio	neutrophil-to-lymphocyte ratio	GCST90056182	3x10-16	NA	0.00007096878967194706	PICS	PICS fine-mapped credible set based on reported top hit	LRRC37A	0.4830050766468048	2173
Mono C	KANSL1-AS1	rs56328224	17_46255427_G_A	Neutrophill count (UKB data field 30140)	neutrophil count	GCST90310174	1.2050000429153442x10-12	0.02774	0.00021233068503705903	PICS	PICS fine-mapped credible set extracted from summary statistics	LRRC37A	0.3021009564399719	2725
Mono C	KANSL1-AS1	rs56328224	17_45948522_C_A	Lymphocyte percentage (UKB data field 30180)	lymphocyte percentage of leukocytes	GCST90309876	3.114000082015991x10-13	0.0295712	0.0002541329115466672	PICS	PICS fine-mapped credible set extracted from summary statistics	MAPT	0.41726210713386536	2724
CD4 ET	KANSL1-AS1	rs55974014	17_45591487_A_G	neutrophil (fraction, minimum, inv-norm transformed)	neutrophil count	GCST90475541	8.09169864654541x10-24	0.014084584748667382	0.0002005395485717409	SuSiE-inf	SuSiE fine-mapped credible set with out-of-sample LD	CRHR1	0.5099560618400574	2395
CD4 ET	KANSL1-AS1	rs55974014	17_45618793_A_T	Sum basophil neutrophil counts	sum of basophil and neutrophil counts	GCST004620	2.3425774574279785x10-11	0.016323855245078723	0.0003559971444392398	SuSiE-inf	SuSiE fine-mapped credible set with out-of-sample LD	MAPT	0.25818219780921936	1733
CD4 ET	KANSL1-AS1	rs55974014	17_45618793_A_T	Neutrophil count	neutrophil count	GCST004629	2.937805652618408x10-11	0.015872561504500494	0.00033554318676241396	SuSiE-inf	SuSiE fine-mapped credible set with out-of-sample LD	MAPT	0.17867916822433472	1809
CD4 ET	KANSL1-AS1	rs55974014	17_45768114_T_C	lymphocyte (absolute count, minimum, inv-norm transformed)	lymphocyte count	GCST90475429	2.378000020980835x10-10	0.02187	0.0002474908955587464	PICS	PICS fine-mapped credible set extracted from summary statistics	MAPT	0.32434797286987305	2811
CD4 ET	KANSL1-AS1	rs55974014	17_45768114_T_C	Lymphocyte count	lymphocyte count	GCST90002316	2.9720447063446045x10-14	0.008773098251850348	0.0005535750550092118	SuSiE-inf	SuSiE fine-mapped credible set with out-of-sample LD	MAPT	0.2875947654247284	1514
CD4 ET	KANSL1-AS1	rs55974014	17_45768114_T_C	lymphocyte (absolute count, minimum, inv-norm transformed)	lymphocyte count	GCST90479665	6.980000019073486x10-9	0.01876	0.00025599920822647385	PICS	PICS fine-mapped credible set extracted from summary statistics	MAPT	0.34357011318206787	2881
CD4 ET	KANSL1-AS1	rs55974014	17_45770203_TTTTGTTTG_T	Lymphocyte count	lymphocyte count	GCST90002388	3.5953187942504883x10-11	-0.00763271	0.0006012525322572461	SuSiE-inf	SuSiE fine-mapped credible set with out-of-sample LD	MAPT	0.21399348974227905	2506
CD4 ET	KANSL1-AS1	rs55974014	17_45849924_G_A	Eosinophil counts	eosinophil count	GCST90134621	1x10-17	-0.038	0.0002475008017884273	PICS	PICS fine-mapped credible set based on reported top hit	SPPL2C	0.3401679992675781	2616
CD4 ET	KANSL1-AS1	rs55974014	17_45941153_T_C	lymphocyte (fraction, maximum, inv-norm transformed)	lymphocyte count	GCST90475432	4.342451095581055x10-15	-0.013841055	0.00047302120090526216	SuSiE-inf	SuSiE fine-mapped credible set with out-of-sample LD	MAPT	0.3556195795536041	2061
CD4 ET	KANSL1-AS1	rs55974014	17_45720994_A_G	Eosinophil counts	eosinophil count	GCST90056180	4x10-14	NA	0.0002265119037909917	PICS	PICS fine-mapped credible set based on reported top hit	MAPT	0.1859586089849472	2682
CD4 ET	KANSL1-AS1	rs55974014	17_46112492_G_A	Lymphocyte percentage of white cells	lymphocyte percentage of leukocytes	GCST004632	5.456538200378418x10-13	-0.017725684	0.00014278736202507608	SuSiE-inf	SuSiE fine-mapped credible set with out-of-sample LD	STH	0.3389870524406433	1707
CD4 ET	KANSL1-AS1	rs55974014	17_46138530_C_T	Neutrophil percentage of white cells	neutrophil percentage of leukocytes	GCST004633	9.970807075500488x10-15	0.018819767160248347	0.000269327591756285	SuSiE-inf	SuSiE fine-mapped credible set with out-of-sample LD	STH	0.4624694883823395	1535
CD4 ET	KANSL1-AS1	rs55974014	17_45948522_C_A	Lymphocyte percentage (UKB data field 30180)	lymphocyte percentage of leukocytes	GCST90309876	3.114000082015991x10-13	0.0295712	0.00021298239072317518	PICS	PICS fine-mapped credible set extracted from summary statistics	MAPT	0.41726210713386536	2724
CD8 NC	KANSL1-AS1	rs383241	17_45386127_A_C	neutrophil (absolute count, mean, inv-norm transformed)	neutrophil count	GCST90475529	9.381999969482422x10-10	0.02334	0.0002142100008325427 7	PICS	PICS fine-mapped credible set extracted from summary statistics	ARHGAP27	0.26441600918769836	2808
CD8 NC	KANSL1-AS1	rs383241	17_45443309_T_C	Neutrophill count (UKB data field 30140)	neutrophil count	GCST90013984	7.261449337005615x10-19	0.010323402122700998	0.0001827527094934136 7	SuSiE-inf	SuSiE fine-mapped credible set with out-of-sample LD	ARHGAP27	0.33282899856567383	2186
CD8 NC	KANSL1-AS1	rs383241	17_45578810_C_A	Eosinophil counts	eosinophil count	GCST90002381	3.2913196086883545x10-38	-0.020678356	0.0003504306215261313	SuSiE-inf	SuSiE fine-mapped credible set with out-of-sample LD	MAPT	0.288733571767807	2234
CD8 NC	KANSL1-AS1	rs383241	17_45447800_C_CT	Neutrophil count	neutrophil count	GCST90002398	6.910816192626953x10-19	0.010611617230070865	0.0003279604476390492	SuSiE-inf	SuSiE fine-mapped credible set with out-of-sample LD	SPPL2C	0.14334917068481445	2494
CD8 NC	KANSL1-AS1	rs383241	17_45591487_A_G	neutrophil (fraction, minimum, inv-norm transformed)	neutrophil count	GCST90475541	8.09169864654541x10-24	0.014084584748667382	0.00019317637338652836	SuSiE-inf	SuSiE fine-mapped credible set with out-of-sample LD	CRHR1	0.5099560618400574	2395
CD8 NC	KANSL1-AS1	rs383241	17_45591486_C_T	lymphocyte (fraction, minimum, inv-norm transformed)	lymphocyte count	GCST90475438	5.3282389640808105x10-13	-0.01288517	0.00024254123266547243	SuSiE-inf	SuSiE fine-mapped credible set with out-of-sample LD	MAPT	0.3223322033882141	2303
CD8 NC	KANSL1-AS1	rs383241	17_45604405_C_T	lymphocyte (absolute count, mean, inv-norm transformed)	lymphocyte count	GCST90475426	7.567112922668457x10-9	-0.011358674	0.0003675572818603256	SuSiE-inf	SuSiE fine-mapped credible set with out-of-sample LD	MAPT	0.194302037358284	2401
CD8 NC	KANSL1-AS1	rs383241	17_45618793_A_T	Sum basophil neutrophil counts	sum of basophil and neutrophil counts	GCST004620	2.3425774574279785x10-11	0.016323855245078723	0.00037653968226813176	SuSiE-inf	SuSiE fine-mapped credible set with out-of-sample LD	MAPT	0.25818219780921936	1733
CD8 NC	KANSL1-AS1	rs383241	17_45618793_A_T	Neutrophil count	neutrophil count	GCST004629	2.937805652618408x10-11	0.015872561504500494	0.0003612360270883155	SuSiE-inf	SuSiE fine-mapped credible set with out-of-sample LD	MAPT	0.17867916822433472	1809
CD8 NC	KANSL1-AS1	rs383241	17_45768114_T_C	lymphocyte (absolute count, minimum, inv-norm transformed)	lymphocyte count	GCST90475429	2.378000020980835x10-10	0.02187	0.0003605761065647562	PICS	PICS fine-mapped credible set extracted from summary statistics	MAPT	0.32434797286987305	2811
CD8 NC	KANSL1-AS1	rs383241	17_45768114_T_C	Lymphocyte count	lymphocyte count	GCST90002316	2.9720447063446045x10-14	0.008773098251850348	0.00031037713299010507	SuSiE-inf	SuSiE fine-mapped credible set with out-of-sample LD	MAPT	0.2875947654247284	1514
CD8 NC	KANSL1-AS1	rs383241	17_45768114_T_C	lymphocyte (fraction, mean, inv-norm transformed)	lymphocyte count	GCST90475435	4.9070000648498535x10-18	0.02716	0.0003621850361179483	PICS	PICS fine-mapped credible set extracted from summary statistics	MAPT	0.2943761944770813	2612
CD8 NC	KANSL1-AS1	rs383241	17_45768114_T_C	lymphocyte (absolute count, minimum, inv-norm transformed)	lymphocyte count	GCST90479665	6.980000019073486x10-9	0.01876	0.0003545420571686623	PICS	PICS fine-mapped credible set extracted from summary statistics	MAPT	0.34357011318206787	2881
CD8 NC	KANSL1-AS1	rs383241	17_45770203_TTTTGTTTG_T	Lymphocyte count	lymphocyte count	GCST90002388	3.5953187942504883x10-11	-0.00763271	0.00030140991856762175	SuSiE-inf	SuSiE fine-mapped credible set with out-of-sample LD	MAPT	0.21399348974227905	2506
CD8 NC	KANSL1-AS1	rs383241	17_45663199_G_T	eosinophil (absolute count, maximum, inv-norm transformed)	eosinophil count	GCST90475288	4.694210052490234x10-28	0.01834665242482715	0.00012268822629946024	SuSiE-inf	SuSiE fine-mapped credible set with out-of-sample LD	CRHR1	0.30067071318626404	338
CD8 NC	KANSL1-AS1	rs383241	17_45855432_G_T	Neutrophill count (UKB data field 30140)	neutrophil count	GCST90013984	2.8343358039855957x10-18	0.010853355122281626	0.0004592624309812844	SuSiE-inf	SuSiE fine-mapped credible set with out-of-sample LD	SPPL2C	0.3202141225337982	2531
CD8 NC	KANSL1-AS1	rs383241	17_45899318_T_C	Granulocyte count	granulocyte count	GCST004614	2.0250000953674316x10-9	0.02259961	0.00024288618731798097	PICS	PICS fine-mapped credible set extracted from summary statistics	MAPT	0.5094848275184631	2497
CD8 NC	KANSL1-AS1	rs383241	17_45899318_T_C	Myeloid white cell count	myeloid leukocyte count	GCST004626	9.201000213623047x10-10	0.02313403	0.0002273640429277843	PICS	PICS fine-mapped credible set extracted from summary statistics	MAPT	0.5226526856422424	2476
CD8 NC	KANSL1-AS1	rs383241	17_45899318_T_C	Sum neutrophil eosinophil counts	sum of neutrophil and eosinophil counts	GCST004613	2.9509999752044678x10-9	0.02233337	0.000250032208513739	PICS	PICS fine-mapped credible set extracted from summary statistics	MAPT	0.4793684184551239	2506
CD8 NC	KANSL1-AS1	rs383241	17_45849924_G_A	Eosinophil counts	eosinophil count	GCST90134621	1x10-17	-0.038	0.00040163967409460107	PICS	PICS fine-mapped credible set based on reported top hit	SPPL2C	0.3401679992675781	2616
CD8 NC	KANSL1-AS1	rs383241	17_45941033_A_G	Eosinophil counts	eosinophil count	GCST007065	3x10-29	NA	0.0003912926682127973 3	PICS	PICS fine-mapped credible set based on reported top hit	MAPT	0.4016060531139374	2511
CD8 NC	KANSL1-AS1	rs383241	17_45941153_T_C	lymphocyte (fraction, maximum, inv-norm transformed)	lymphocyte count	GCST90475432	4.342451095581055x10-15	-0.013841055	0.0003474868112532439	SuSiE-inf	SuSiE fine-mapped credible set with out-of-sample LD	MAPT	0.3556195795536041	2061
CD8 NC	KANSL1-AS1	rs383241	17_45847931_C_T	neutrophil (fraction, maximum, inv-norm transformed)	neutrophil count	GCST90479713	7.110000133514404x10-18	0.02941	0.0003756495106094371	PICS	PICS fine-mapped credible set extracted from summary statistics	SPPL2C	0.3140776753425598	2621
CD8 NC	KANSL1-AS1	rs383241	17_45948522_C_A	Neutrophill percentage (UKB data field 30200)	neutrophil percentage of leukocytes	GCST90309878	6.493000030517578x10-15	-0.0318963	0.00036967300101574597	PICS	PICS fine-mapped credible set extracted from summary statistics	MAPT	0.41726210713386536	2685
CD8 NC	KANSL1-AS1	rs383241	17_45720994_A_G	Eosinophil counts	eosinophil count	GCST90056180	4x10-14	NA	0.00040265752421735345	PICS	PICS fine-mapped credible set based on reported top hit	MAPT	0.1859586089849472	2682
CD8 NC	KANSL1-AS1	rs383241	17_45745406_T_C	Eosinophil percentage of white cells	eosinophil percentage of leukocytes	GCST90002382	8.442001342773438x10-49	-0.022914564	0.001263056154789197	SuSiE-inf	SuSiE fine-mapped credible set with out-of-sample LD	CRHR1	0.30252769589424133	851
CD8 NC	KANSL1-AS1	rs383241	17_46257908_G_A	Osteoarthritis; localized (PheCode 740.1)	osteoarthritis	GCST90044591	1.341586947441101x10-9	0.008216219391190696	0.0003474281631496668	SuSiE-inf	SuSiE fine-mapped credible set with out-of-sample LD	MAPT	0.3438669443130493	2224
CD8 NC	KANSL1-AS1	rs383241	17_46260158_A_T	Obstructive chronic bronchitis (PheCode 496.21)	chronic bronchitis	GCST90476030	2.7960000038146973x10-8	-0.007748819	0.0003123824771907011	SuSiE-inf	SuSiE fine-mapped credible set with out-of-sample LD	STH	0.3092050552368164	2548
CD8 NC	KANSL1-AS1	rs383241	17_45980229_G_A	Superficial cellulitis and abscess (PheCode 681)	cellulitis, abscess	GCST90476165	7.431000232696533x10-11	0.009585597357696183	0.00014338559010022335	SuSiE-inf	SuSiE fine-mapped credible set with out-of-sample LD	MAPT	0.16386571526527405	2473
CD8 NC	KANSL1-AS1	rs383241	17_46036724_CT_C	eosinophil (fraction, minimum, inv-norm transformed)	eosinophil count	GCST90475303	3.4830000400543213x10-28	-0.03702	0.0003712665017406246	PICS	PICS fine-mapped credible set extracted from summary statistics	MAPT	0.2499091923236847	2593
CD8 NC	KANSL1-AS1	rs383241	17_46036724_CT_C	eosinophil (fraction, mean, inv-norm transformed)	eosinophil count	GCST90475300	6.54855489730835x10-49	-0.023639791	0.00023478772746125774	SuSiE-inf	SuSiE fine-mapped credible set with out-of-sample LD	MAPT	0.2379968911409378	2090
CD8 NC	KANSL1-AS1	rs383241	17_46042134_C_CTT	Neutrophils and lymphocytes in blood (confirmatory factor anal	neutrophil measurement, lymphocyte amount	GCST90309369	2.139305591583252x10-23	0.01648682789443355	0.0003544009601268649	SuSiE-inf	SuSiE fine-mapped credible set with out-of-sample LD	MAPT	0.23639625310897827	2271
CD8 NC	KANSL1-AS1	rs383241	17_46138530_C_T	Neutrophil percentage of white cells	neutrophil percentage of leukocytes	GCST004633	9.970807075500488x10-15	0.018819767160248347	0.000248313139771076	SuSiE-inf	SuSiE fine-mapped credible set with out-of-sample LD	STH	0.4624694883823395	1535
CD8 NC	KANSL1-AS1	rs383241	17_45989494_C_CA	Neutrophil percentage of white cells	neutrophil percentage of leukocytes	GCST90002399	3.2710328102111816x10-29	0.013834058143875567	0.00036876030647178587	SuSiE-inf	SuSiE fine-mapped credible set with out-of-sample LD	MAPT	0.20078477263450623	2517

Cell type	Gene	SNP	LeadVariant	Trait	Phenotype	Study	P-value	Beta	PosteriorProbability	FineMappingMethod	FineMappingConfidence	TopL2G	l2GScore	CredibleSetSize
CD8 NC	KANSL1-AS1	rs383241	17_46005715_G_A	eosinophil (fraction, mean, inv-norm transformed)	eosinophil count	GCST90479605	2.4800000190734863x10-31	-0.04771	0.00041000102136469767	PICS	PICS fine-mapped credible set extracted from summary statistics	MAPT	0.3352709114551544	2467
CD8 NC	KANSL1-AS1	rs383241	17_46211866_G_T	Eosinophil count (UKB data field 30150)	eosinophil count	GCST90013985	2.6277098655700684x10-36	-0.019559268	0.00033816894512815366	SuSiE-inf	SuSiE fine-mapped credible set with out-of-sample LD	MAPT	0.23589292168617249	2258
CD8 NC	KANSL1-AS1	rs383241	17_46236156_G_T	Lymphocyte-to-monocyte ratio	lymphocyte:monocyte ratio	GCST90056181	1x10-19	NA	0.00039882365598073504	PICS	PICS fine-mapped credible set based on reported top hit	STH	0.28819653391838074	2579
CD8 NC	KANSL1-AS1	rs383241	17_46202437_T_G	Osteoarthrosis (PheCode 740)	osteoarthritis	GCST90436719	6.05502986907959x10-11	0.009844912896258315	0.0004104415982179102	SuSiE-inf	SuSiE fine-mapped credible set with out-of-sample LD	KANSL1	0.2537720203399658	1895
CD8 NC	KANSL1-AS1	rs383241	17_46246819_A_G	Lymphocyte percentage of white cells	lymphocyte percentage of leukocytes	GCST90002389	1.1399171352386475x10-27	-0.016283486	0.00030322441538483167	SuSiE-inf	SuSiE fine-mapped credible set with out-of-sample LD	STH	0.27214905619621277	2404
CD8 NC	KANSL1-AS1	rs383241	17_46255427_G_A	Neutrophil count	neutrophil count	GCST90002398	7.797736644744873x10-21	0.011998169058646207	0.0004439718321790771	SuSiE-inf	SuSiE fine-mapped credible set with out-of-sample LD	STH	0.3092050552368164	2485
CD8 NC	KANSL1-AS1	rs383241	17_46255427_G_A	Neutrophil-to-lymphocyte ratio	neutrophil-to-lymphocyte ratio	GCST90056182	3x10-16	NA	0.00007153376225567044	PICS	PICS fine-mapped credible set based on reported top hit	LRRC37A	0.4830050766468048	2173
CD8 NC	KANSL1-AS1	rs383241	17_46255427_G_A	Neutrophill count (UKB data field 30140)	neutrophil count	GCST90310174	1.2050000429153442x10-12	0.02774	0.00021364680635695062	PICS	PICS fine-mapped credible set extracted from summary statistics	LRRC37A	0.3021009564399719	2725
CD8 NC	KANSL1-AS1	rs383241	17_45948522_C_A	Lymphocyte percentage (UKB data field 30180)	lymphocyte percentage of leukocytes	GCST90309876	3.114000082015991x10-13	0.0295712	0.0003653375089531801	PICS	PICS fine-mapped credible set extracted from summary statistics	MAPT	0.41726210713386536	2724
CD8 NC	KANSL1-AS1	rs383241	17_46504918_T_G	eosinophil (fraction, maximum, inv-norm transformed)	eosinophil count	GCST90475297	1.7489999532699585x10-33	-0.04235	0.00005893525996491448	PICS	PICS fine-mapped credible set extracted from summary statistics	LRRC37A2	0.546727180480957	1593
CD4 NC	PM20D1	rs1772143	1_205813590_A_G	Monocyte count (UKB data field 30130)	monocyte count	GCST90013983	7.113784313201904x10-17	0.01278628709033188	0.26312822044791034	SuSiE-inf	SuSiE fine-mapped credible set with out-of-sample LD	SLC41A1	0.8142264485359192	8
CD4 NC	PM20D1	rs1772143	1_205813590_A_G	Monocyte count	monocyte count	GCST90002393	2.4259190559387207x10-19	0.013769547947875292	0.2740776731336341	SuSiE-inf	SuSiE fine-mapped credible set with out-of-sample LD	SLC41A1	0.811953604221344	7
CD4 NC	PM20D1	rs1772143	1_205774297_A_C	Monocyte percentage of white cells	monocyte percentage of leukocytes	GCST004609	4.3682475090026855x10-10	0.014384592506590804	0.026149986935968164	SuSiE-inf	SuSiE fine-mapped credible set with out-of-sample LD	PM20D1	0.6988953948020935	16
CD4 NC	PM20D1	rs1772143	1_205813590_A_G	Monocyte count	monocyte count	GCST90018967	6.695000171661377x10-13	0.0169	0.05831113315361791	PICS	PICS fine-mapped credible set extracted from summary statistics	SLC41A1	0.7958654165267944	22
CD4 NC	PM20D1	rs1772143	1_205813590_A_G	Monocyte count	monocyte count	GCST90002344	2x10-22	NA	0.04218441588650992 4	PICS	PICS fine-mapped credible set based on reported top hit	PM20D1	0.8147656321525574	6
CD4 NC	PM20D1	rs1772143	1_205817807_T_C	monocyte (fraction, mean, inv-norm transformed)	monocyte count	GCST90475511	1.1130627393722534x10-12	0.012712373612674988	0.1455215018763822	SuSiE-inf	SuSiE fine-mapped credible set with out-of-sample LD	SLC41A1	0.8123235106468201	12
CD4 NC	PM20D1	rs1772143	1_205830859_T_A	Monocyte count	monocyte count	GCST90002340	7.4264984130859375x10-26	0.014324325755776753	0.37830070348053524	SuSiE-inf	SuSiE fine-mapped credible set with out-of-sample LD	PM20D1	0.8849183917045593	5
CD4 NC	PM20D1	rs1772143	1_205798757_G_A	Monocyte count	monocyte count	GCST004625	1.1399197578430176x10-10	0.014910835604713991	0.07556675535010783	SuSiE-inf	SuSiE fine-mapped credible set with out-of-sample LD	SLC41A1	0.8608401417732239	8
CD4 NC	PM20D1	rs1772143	1_205805711_T_C	monocyte (absolute count, mean, inv-norm transformed)	monocyte count	GCST90475502	6.235194206237793x10-10	0.012443658314790182	0.03792534840447073	SuSiE-inf	SuSiE fine-mapped credible set with out-of-sample LD	SLC41A1	0.755103349685669	19
CD4 NC	PM20D1	rs9438393	1_205813590_A_G	Monocyte count (UKB data field 30130)	monocyte count	GCST90013983	7.113784313201904x10-17	0.01278628709033188	0.3258148554459584	SuSiE-inf	SuSiE fine-mapped credible set with out-of-sample LD	SLC41A1	0.8142264485359192	8
CD4 NC	PM20D1	rs9438393	1_205813590_A_G	Monocyte count	monocyte count	GCST90002393	2.4259190559387207x10-19	0.013769547947875292	0.36781574494320274	SuSiE-inf	SuSiE fine-mapped credible set with out-of-sample LD	SLC41A1	0.811953604221344	7
CD4 NC	PM20D1	rs9438393	1_205774297_A_C	Monocyte percentage of white cells	monocyte percentage of leukocytes	GCST004609	4.3682475090026855x10-10	0.014384592506590804	0.036369959610105085	SuSiE-inf	SuSiE fine-mapped credible set with out-of-sample LD	PM20D1	0.6988953948020935	16
CD4 NC	PM20D1	rs9438393	1_205813590_A_G	Monocyte count	monocyte count	GCST90018967	6.695000171661377x10-13	0.0169	0.23290567386790198	PICS	PICS fine-mapped credible set extracted from summary statistics	SLC41A1	0.7958654165267944	22
CD4 NC	PM20D1	rs9438393	1_205813590_A_G	Monocyte count	monocyte count	GCST90002344	2x10-22	NA	0.5888105657652105	PICS	PICS fine-mapped credible set based on reported top hit	PM20D1	0.8147656321525574	6
CD4 NC	PM20D1	rs9438393	1_205817807_T_C	monocyte (fraction, mean, inv-norm transformed)	monocyte count	GCST90475511	1.1130627393722534x10-12	0.012712373612674988	0.12303524581733641	SuSiE-inf	SuSiE fine-mapped credible set with out-of-sample LD	SLC41A1	0.8123235106468201	12
CD4 NC	PM20D1	rs9438393	1_205798757_G_A	Monocyte count	monocyte count	GCST004625	1.1399197578430176x10-10	0.014910835604713991	0.12868768842243763	SuSiE-inf	SuSiE fine-mapped credible set with out-of-sample LD	SLC41A1	0.8608401417732239	8
CD4 NC	PM20D1	rs9438393	1_205805711_T_C	monocyte (absolute count, mean, inv-norm transformed)	monocyte count	GCST90475502	6.235194206237793x10-10	0.012443658314790182	0.13496255008118882	SuSiE-inf	SuSiE fine-mapped credible set with out-of-sample LD	SLC41A1	0.755103349685669	19
CD4 NC	PM20D1	rs823080	1_205813590_A_G	Monocyte count (UKB data field 30130)	monocyte count	GCST90013983	7.113784313201904x10-17	0.01278628709033188	0.1819460231614163	SuSiE-inf	SuSiE fine-mapped credible set with out-of-sample LD	SLC41A1	0.8142264485359192	8
CD4 NC	PM20D1	rs823080	1_205813590_A_G	Monocyte count	monocyte count	GCST90002393	2.4259190559387207x10-19	0.013769547947875292	0.18690810906887395	SuSiE-inf	SuSiE fine-mapped credible set with out-of-sample LD	SLC41A1	0.811953604221344	7
CD4 NC	PM20D1	rs823080	1_205774297_A_C	Monocyte percentage of white cells	monocyte percentage of leukocytes	GCST004609	4.3682475090026855x10-10	0.014384592506590804	0.015764252573298623	SuSiE-inf	SuSiE fine-mapped credible set with out-of-sample LD	PM20D1	0.6988953948020935	16
CD4 NC	PM20D1	rs823080	1_205813590_A_G	Monocyte count	monocyte count	GCST90018967	6.695000171661377x10-13	0.0169	0.13296987001897173	PICS	PICS fine-mapped credible set extracted from summary statistics	SLC41A1	0.7958654165267944	22
CD4 NC	PM20D1	rs823080	1_205813590_A_G	Monocyte count	monocyte count	GCST90002344	2x10-22	NA	0.2443415974405839	PICS	PICS fine-mapped credible set based on reported top hit	PM20D1	0.8147656321525574	6
CD4 NC	PM20D1	rs823080	1_205817807_T_C	monocyte (fraction, mean, inv-norm transformed)	monocyte count	GCST90475511	1.1130627393722534x10-12	0.012712373612674988	0.1047937768030997	SuSiE-inf	SuSiE fine-mapped credible set with out-of-sample LD	SLC41A1	0.8123235106468201	12
CD4 NC	PM20D1	rs823080	1_205830859_T_A	Monocyte count	monocyte count	GCST90002340	7.4264984130859375x10-26	0.014324325755776753	0.1822958985751251	SuSiE-inf	SuSiE fine-mapped credible set with out-of-sample LD	PM20D1	0.8849183917045593	5
CD4 NC	PM20D1	rs823080	1_205798757_G_A	Monocyte count	monocyte count	GCST004625	1.1399197578430176x10-10	0.014910835604713991	0.08805414545544682	SuSiE-inf	SuSiE fine-mapped credible set with out-of-sample LD	SLC41A1	0.8608401417732239	8
CD4 NC	PM20D1	rs823080	1_205805711_T_C	monocyte (absolute count, mean, inv-norm transformed)	monocyte count	GCST90475502	6.235194206237793x10-10	0.012443658314790182	0.120428069391181	SuSiE-inf	SuSiE fine-mapped credible set with out-of-sample LD	SLC41A1	0.755103349685669	19
CD4 NC	PM20D1	rs708727	1_205813590_A_G	Monocyte count (UKB data field 30130)	monocyte count	GCST90013983	7.113784313201904x10-17	0.01278628709033188	0.05942705194931575	SuSiE-inf	SuSiE fine-mapped credible set with out-of-sample LD	SLC41A1	0.8142264485359192	8
CD4 NC	PM20D1	rs708727	1_205813590_A_G	Monocyte count	monocyte count	GCST90002393	2.4259190559387207x10-19	0.013769547947875292	0.04233068872619285	SuSiE-inf	SuSiE fine-mapped credible set with out-of-sample LD	SLC41A1	0.811953604221344	7
CD4 NC	PM20D1	rs708727	1_205774297_A_C	Monocyte percentage of white cells	monocyte percentage of leukocytes	GCST004609	4.3682475090026855x10-10	0.014384592506590804	0.07170516743537315	SuSiE-inf	SuSiE fine-mapped credible set with out-of-sample LD	PM20D1	0.6988953948020935	16
CD4 NC	PM20D1	rs708727	1_205813590_A_G	Monocyte count	monocyte count	GCST90018967	6.695000171661377x10-13	0.0169	0.10117032856619036	PICS	PICS fine-mapped credible set extracted from summary statistics	SLC41A1	0.7958654165267944	22
CD4 NC	PM20D1	rs708727	1_205813590_A_G	Monocyte count	monocyte count	GCST90002344	2x10-22	NA	0.026079618135703607	PICS	PICS fine-mapped credible set based on reported top hit	PM20D1	0.8147656321525574	6
CD4 NC	PM20D1	rs708727	1_205817807_T_C	monocyte (fraction, mean, inv-norm transformed)	monocyte count	GCST90475511	1.1130627393722534x10-12	0.012712373612674988	0.050020350036563146	SuSiE-inf	SuSiE fine-mapped credible set with out-of-sample LD	SLC41A1	0.8123235106468201	12
CD4 NC	PM20D1	rs708727	1_205830859_T_A	Monocyte count	monocyte count	GCST90002340	7.4264984130859375x10-26	0.014324325755776753	0.023191476544330777	SuSiE-inf	SuSiE fine-mapped credible set with out-of-sample LD	PM20D1	0.8849183917045593	5
CD4 NC	PM20D1	rs708727	1_205798757_G_A	Monocyte count	monocyte count	GCST004625	1.1399197578430176x10-10	0.014910835604713991	0.16892941555381918	SuSiE-inf	SuSiE fine-mapped credible set with out-of-sample LD	SLC41A1	0.8608401417732239	8
CD4 NC	PM20D1	rs708727	1_205805711_T_C	monocyte (absolute count, mean, inv-norm transformed)	monocyte count	GCST90475502	6.235194206237793x10-10	0.012443658314790182	0.137979688654376	SuSiE-inf	SuSiE fine-mapped credible set with out-of-sample LD	SLC41A1	0.755103349685669	19
CD4 NC	PM20D1	rs823074	1_205813590_A_G	Monocyte count (UKB data field 30130)	monocyte count	GCST90013983	7.113784313201904x10-17	0.01278628709033188	0.059856857880687106	SuSiE-inf	SuSiE fine-mapped credible set with out-of-sample LD	SLC41A1	0.8142264485359192	8
CD4 NC	PM20D1	rs823074	1_205813590_A_G	Monocyte count	monocyte count	GCST90002393	2.4259190559387207x10-19	0.013769547947875292	0.039734257504348273	SuSiE-inf	SuSiE fine-mapped credible set with out-of-sample LD	SLC41A1	0.811953604221344	7
CD4 NC	PM20D1	rs823074	1_205774297_A_C	Monocyte percentage of white cells	monocyte percentage of leukocytes	GCST004609	4.3682475090026855x10-10	0.014384592506590804	0.03519636869549766	SuSiE-inf	SuSiE fine-mapped credible set with out-of-sample LD	PM20D1	0.6988953948020935	16
CD4 NC	PM20D1	rs823074	1_205813590_A_G	Monocyte count	monocyte count	GCST90018967	6.695000171661377x10-13	0.0169	0.10501531600246203	PICS	PICS fine-mapped credible set extracted from summary statistics	SLC41A1	0.7958654165267944	22
CD4 NC	PM20D1	rs823074	1_205817807_T_C	monocyte (fraction, mean, inv-norm transformed)	monocyte count	GCST90475511	1.1130627393722534x10-12	0.012712373612674988	0.05805836336141849	SuSiE-inf	SuSiE fine-mapped credible set with out-of-sample LD	SLC41A1	0.8123235106468201	12
CD4 NC	PM20D1	rs823074	1_205830859_T_A	Monocyte count	monocyte count	GCST90002340	7.4264984130859375x10-26	0.014324325755776753	0.029031451744951805	SuSiE-inf	SuSiE fine-mapped credible set with out-of-sample LD	PM20D1	0.8849183917045593	5
CD4 NC	PM20D1	rs823074	1_205798757_G_A	Monocyte count	monocyte count	GCST004625	1.1399197578430176x10-10	0.014910835604713991	0.15003062172358617	SuSiE-inf	SuSiE fine-mapped credible set with out-of-sample LD	SLC41A1	0.8608401417732239	8
CD4 NC	PM20D1	rs823074	1_205805711_T_C	monocyte (absolute count, mean, inv-norm transformed)	monocyte count	GCST90475502	6.235194206237793x10-10	0.012443658314790182	0.1735346776182318	SuSiE-inf	SuSiE fine-mapped credible set with out-of-sample LD	SLC41A1	0.755103349685669	19
CD4 NC	CNN2	rs4807455	19_1044262_T_A	neutrophil (absolute count, mean, inv-norm transformed)	neutrophil count	GCST90479711	1.41997492313385x10-11	-0.010527709	0.04976802327571602	SuSiE-inf	SuSiE fine-mapped credible set with out-of-sample LD	ABCA7	0.3545154929161072	4
CD4 NC	CNN2	rs4807455	19_1033560_G_A	neutrophil (absolute count, minimum, inv-norm transformed)	neutrophil count	GCST90475532	2.13700008392334x10-12	-0.02536	0.9747741834430768	PICS	PICS fine-mapped credible set extracted from summary statistics	CNN2	0.47906729578971863	1
CD4 NC	CNN2	rs4807455	19_1033560_G_A	neutrophil (absolute count, minimum, inv-norm transformed)	neutrophil count	GCST90479712	3.814920425415039x10-13	-0.011433939	0.9823675936165127	SuSiE-inf	SuSiE fine-mapped credible set with out-of-sample LD	CNN2	0.47245049476623535	1
CD4 NC	CNN2	rs7246841	19_1030432_T_C	Eosinophil percentage of white cells	eosinophil percentage of leukocytes	GCST90002382	2.93390154838562x10-13	-0.014442901	0.7078869249926534	SuSiE-inf	SuSiE fine-mapped credible set with out-of-sample LD	CNN2	0.7803710699081421	2
CD4 NC	CNN2	rs7246841	19_1030651_C_G	Eosinophil count (UKB data field 30150)	eosinophil count	GCST90013985	6.5486555099487305x10-6	-0.012857872	0.47029973413477855	SuSiE-inf	SuSiE fine-mapped credible set with out-of-sample LD	CNN2	0.7881802320480347	2
CD4 NC	CNN2	rs7246841	19_1030651_C_G	eosinophil (fraction, mean, inv-norm transformed)	eosinophil count	GCST90475300	5.61945915222168x10-11	-0.015980612	0.3649826429682483	SuSiE-inf	SuSiE fine-mapped credible set with out-of-sample LD	CNN2	0.7803710699081421	2
CD4 NC	CNN2	rs10419072	19_1030767_G_A	neutrophil (absolute count, minimum, inv-norm transformed)	neutrophil count	GCST90475531	7.7634758949279785x10-9	0.020708124647778638	0.4284886186482824	SuSiE-inf	SuSiE fine-mapped credible set with out-of-sample LD	CNN2	0.6326821446418762	3
CD8 ET	B4GALNT3	rs7314691	12_553672_T_C	Circulating plasma alpha-Klotho levels	circulating alpha-Klotho measurement	GCST90091247	5.703173637390137x10-15	0.10785529175403627	0.32805353322931374	SuSiE-inf	SuSiE fine-mapped credible set with out-of-sample LD	B4GALNT3	0.896360456943512	3
CD8 ET	B4GALNT3	rs7314691	12_553672_T_C	Circulating plasma alpha-Klotho levels	circulating alpha-Klotho measurement	GCST90091246	1.6181325912475586x10-14	0.10914420406565589	0.37927401686924017	SuSiE-inf	SuSiE fine-mapped credible set with out-of-sample LD	B4GALNT3	0.9337891340255737	2
NK	B4GALNT3	rs7314691	12_553672_T_C	Circulating plasma alpha-Klotho levels	circulating alpha-Klotho measurement	GCST90091247	5.703173637390137x10-15	0.10785529175403627	0.32805353322931374	SuSiE-inf	SuSiE fine-mapped credible set with out-of-sample LD	B4GALNT3	0.896360456943512	3
NK	B4GALNT3	rs7314691	12_553672_T_C	Circulating plasma alpha-Klotho levels	circulating alpha-Klotho measurement	GCST90091246	1.6181325912475586x10-14	0.10914420406565589	0.37927401686924017	SuSiE-inf	SuSiE fine-mapped credible set with out-of-sample LD	B4GALNT3	0.9337891340255737	2
NK	B4GALNT3	rs1056008	12_553672_T_C	Circulating plasma alpha-Klotho levels	circulating alpha-Klotho measurement	GCST90091247	5.703173637390137x10-15	0.10785529175403627	0.5184275448155131	SuSiE-inf	SuSiE fine-mapped credible set with out-of-sample LD	B4GALNT3	0.896360456943512	3
NK	B4GALNT3	rs1056008	12_553672_T_C	Circulating plasma alpha-Klotho levels	circulating alpha-Klotho measurement	GCST90091246	1.6181325912475586x10-14	0.10914420406565589	0.6062314528603661	SuSiE-inf	SuSiE fine-mapped credible set with out-of-sample LD	B4GALNT3	0.9337891340255737	2
NK	B4GALNT3	rs7298766	12_552490_A_G	Protein kinase C-binding protein NELL1 levels	protein kinase C-binding protein NELL1 measurement	GCST90248637	4x10-56	0.226	0.9997996116465802	PICS	PICS fine-mapped credible set based on reported top hit	B4GALNT3	0.9146315455436707	1
NK	B4GALNT3	rs7298766	12_553672_T_C	Circulating plasma alpha-Klotho levels	circulating alpha-Klotho measurement	GCST90091247	5.703173637390137x10-15	0.10785529175403627	0.15269963993862054	SuSiE-inf	SuSiE fine-mapped credible set with out-of-sample LD	B4GALNT3	0.896360456943512	3
