# Supplementary material for: Immune cell-specific genetic architecture of Alzheimer’s disease revealed by multi-omics analysis for therapeutic target discovery and prioritization
Source: Transl Psychiatry. 2026 Jun 23;16:449. doi: 10.1038/s41398-026-04199-9 (PMC13538538; doi:10.1038/s41398-026-04199-9)
Supplement: Supplementary file 12 [file 41398_2026_4199_MOESM9_ESM.pdf]

Table S9. Significant immune/inflammation and neurodegenerative disease trait associations of IVs used in MR analyses from Open Targets Platform.

Cell type	Gene	SNP	LeadVariant	Trait	Phenotype	Study	P-value	Beta	PosteriorProbability	FineMappingMethod	FineMappingConfidence	TopL2G	l2GScore	CredibleSetSize
CD8 S10(	KANSL1-AS1	rs55974014	17_45423221_G_A	Rheumatoid arthritis (PheCode 714.1)	rheumatoid arthritis	GCST90479433	9.100000381469727x10-9	0.009962523781080345	0.00005741124167948369	SuSiE-inf	SuSiE fine-mapped credible set with out-of-sample LD	ARHGAP27	0.4698462188243866	1714
CD8 S10(	KANSL1-AS1	rs55974014	17_45673544_C_T	Parkinson's disease	Parkinson disease	GCST90475830	8.974945068359375x10-13	0.009834181096919671	0.000020853209619890349	SuSiE-inf	SuSiE fine-mapped credible set with out-of-sample LD	SPPL2C	0.26543086767196655	2766
CD8 S10(	KANSL1-AS1	rs55974014	17_45641777_A_G	Parkinson's disease	Parkinson disease	GCST000528	2x10-16	-0.262364264467491	0.000020913456305867196	PICS	PICS fine-mapped credible set based on reported top hit	SPPL2C	0.32658255100250244	2630
CD8 S10(	KANSL1-AS1	rs55974014	17_45641777_A_G	Corticobasal degeneration	corticobasal degeneration disorder	GCST002971	1x10-12	1.308332819650179	0.000022717618926197078	PICS	PICS fine-mapped credible set based on reported top hit	SPPL2C	0.32658255100250244	2705
CD8 S10(	KANSL1-AS1	rs55974014	17_45815607_T_C	Major depressive disorder or hospitalized COVID-19 (pleiotropy)	COVID-19, major depressive disorder	GCST90296444	1x10-12	NA	0.000020374126756260208	PICS	PICS fine-mapped credible set based on reported top hit	STH	0.20836710929870605	2700
CD8 S10(	KANSL1-AS1	rs55974014	17_45645823_G_A	Ovarian cancer or critical COVID-19 (pleiotropy)	ovarian carcinoma, COVID-19	GCST90296492	8x10-16	NA	0.000020443733313407222	PICS	PICS fine-mapped credible set based on reported top hit	SPPL2C	0.24418936669826508	2629
CD8 S10(	KANSL1-AS1	rs55974014	17_45825631_G_A	Major depressive disorder or severe COVID-19 (pleiotropy)	COVID-19, major depressive disorder	GCST90296443	1x10-9	NA	0.000022291182509290025	PICS	PICS fine-mapped credible set based on reported top hit	STH	0.21132484078407288	2799
CD8 S10(	KANSL1-AS1	rs55974014	17_45771384_T_C	Illnesses of mother: Parkinson's disease (UKB data field 20110_11)	Parkinson disease	GCST90042681	6.2085862159729x10-12	-0.0101908146104154	0.000320926516586163	SuSiE-inf	SuSiE fine-mapped credible set with out-of-sample LD	MAPT	0.2560127377510071	2165
CD8 S10(	KANSL1-AS1	rs55974014	17_45913906_A_G	Alzheimer's disease or educational attainment (pleiotropy)	educational attainment, Alzheimer disease	GCST90095190	7x10-9	-43.902805	0.0002220867579296857	PICS	PICS fine-mapped credible set based on reported top hit	MAPT	0.4050951302051544	2846
CD8 S10(	KANSL1-AS1	rs55974014	17_45846317_A_G	Corticobasal degeneration	corticobasal degeneration disorder	GCST002970	1x10-9	1.2412685890696329	0.00021974946423159044	PICS	PICS fine-mapped credible set based on reported top hit	SPPL2C	0.32074519991874695	2800
CD8 S10(	KANSL1-AS1	rs55974014	17_45707983_T_C	COVID-19 (hospitalized vs population)	COVID-19	GCST90134597	4x10-12	-0.0943106794712412	0.0002191129150504864	PICS	PICS fine-mapped credible set based on reported top hit	SPPL2C	0.20194369554519653	2708
CD8 S10(	KANSL1-AS1	rs55974014	17_45707983_T_C	COVID-19 severity	COVID-19 symptoms measurement	GCST90274862	5x10-13	-0.0972	0.00021429110906502394	PICS	PICS fine-mapped credible set based on reported top hit	SPPL2C	0.24951985478401184	2685
CD8 S10(	KANSL1-AS1	rs55974014	17_46049399_C_A	Parkinson's disease	Parkinson disease	GCST009512	9x10-13	-0.311323	0.0002068642814015056	PICS	PICS fine-mapped credible set based on reported top hit	MAPT	0.2455509603023529	2745
CD8 S10(	KANSL1-AS1	rs55974014	17_46110126_A_G	Parkinson's disease	Parkinson disease	GCST002455	8x10-9	0.44468582126144574	0.00022674201377110683	PICS	PICS fine-mapped credible set based on reported top hit	STH	0.37043941020965576	2859
CD8 S10(	KANSL1-AS1	rs55974014	17_46115202_C_T	Parkinson's disease	Parkinson disease	GCST009324	1.0077089071273804x10-12	-0.0418829269594708	0.00023527149029029765	SuSiE-inf	SuSiE fine-mapped credible set with out-of-sample LD	STH	0.3473161458969116	1735
CD8 S10(	KANSL1-AS1	rs55974014	17_46142465_T_A	COVID-19 (hospitalized vs population)	COVID-19	GCST90095124	2x10-10	NA	0.00022519187013155418	PICS	PICS fine-mapped credible set based on reported top hit	STH	0.4211447238922119	2786
CD8 S10(	KANSL1-AS1	rs55974014	17_46142465_T_A	COVID-19 with respiratory failure	respiratory failure, COVID-19	GCST90278684	3x10-11	NA	0.00022064086599139098	PICS	PICS fine-mapped credible set based on reported top hit	STH	0.4211447238922119	2756
CD8 S10(	KANSL1-AS1	rs55974014	17_46003698_A_G	Parkinson's disease	Parkinson disease	GCST000855	7x10-12	0.26236426446749106	0.00020708013233073814	PICS	PICS fine-mapped credible set based on reported top hit	MAPT	0.35486161708831787	2725
CD8 S10(	KANSL1-AS1	rs55974014	17_46024409_A_AT	Artrosis, including avohilmo	osteoarthritis	FINNGEN_R12_M13_ARTHROSIS_INCLAVC	3.6589999198913574x10-10	0.00021432375969401 6	0.000165399281433934	SuSie	SuSiE fine-mapped credible set with in-sample LD	MAPT	0.3083437979221344	2451
CD8 S10(	KANSL1-AS1	rs55974014	17_46152620_T_C	COVID-19 (critical illness vs population or mild symptoms)	COVID-19	GCST90104030	4x10-9	-0.18232155679395 4	0.0002247453472361834	PICS	PICS fine-mapped credible set based on reported top hit	STH	0.2508043050765991	2833
CD8 S10(	KANSL1-AS1	rs55974014	17_46287153_C_G	Parkinson's disease	Parkinson disease	FINNGEN_R12_G6_PARKINSON	5.935999870300293x10-12	-0.00110625964127491	0.00147610531081006	SuSie	SuSiE fine-mapped credible set with in-sample LD	LRRC37A	0.18633273243904114	2488
B Mem	KANSL1-AS1	rs1378358	17_46720553_C_T	Alzheimer's disease	Alzheimer disease	GCST90027158	2.571000099182129x10-9	-0.0593	0.001184188694283257	PICS	PICS fine-mapped credible set extracted from summary sta	STH	0.12728427350521088	2537
B Mem	KANSL1-AS1	rs1378358	17_46024409_A_AT	Artrosis, including avohilmo	osteoarthritis	FINNGEN_R12_M13_ARTHROSIS_INCLAVC	3.6589999198913574x10-10	0.00021432375969401 6	0.00027982109284147	SuSie	SuSiE fine-mapped credible set with in-sample LD	MAPT	0.3083437979221344	2451
B Mem	KANSL1-AS1	rs1378358	17_46024409_A_AT	Dementia	Dementia	FINNGEN_R12_F5_DEMENTIA	1.815999984741211x10-10	-0.0277816419353282	0.000035848633966854	SuSie	SuSiE fine-mapped credible set with in-sample LD	STH	0.10969875752925873	688
B Mem	KANSL1-AS1	rs1378358	17_46024483_T_TC	Unspecific neurodegenerative disorder	neurodegenerative disease	FINNGEN_R12_G6_NEURODEGENERATIVE	7.847000122070312 5x10-10	-0.0263685534837194	0.0000768097082748344	SuSie	SuSiE fine-mapped credible set with in-sample LD	MAPT	0.1823212206363678	900
B Mem	KANSL1-AS1	rs1378358	17_46779275_G_C	Alzheimer's disease	Alzheimer disease	GCST90399479	7x10-9	NA	0.0009511375398963148	PICS	PICS fine-mapped credible set based on reported top hit	STH	0.12728427350521088	2291
B Mem	KANSL1-AS1	rs1378358	17_46779275_G_C	Parkinson's disease	Parkinson disease	GCST001430	3x10-17	NA	0.0016950196282597197	PICS	PICS fine-mapped credible set based on reported top hit	STH	0.12393897026777267	2083
B Mem	KANSL1-AS1	rs1378358	17_46779986_T_C	Breast cancer or critical COVID-19 (pleiotropy)	COVID-19, breast cancer	GCST90296489	4x10-18	NA	0.0018398564823453548	PICS	PICS fine-mapped credible set based on reported top hit	STH	0.12393897026777267	2069
B Mem	KANSL1-AS1	rs1378358	17_46779986_T_C	Ovarian cancer or critical COVID-19 (pleiotropy)	ovarian carcinoma, COVID-19	GCST90296492	3x10-15	NA	0.0015546311399698522	PICS	PICS fine-mapped credible set based on reported top hit	STH	0.12393897026777267	2137
B Mem	KANSL1-AS1	rs1378358	17_46788237_T_C	Alzheimer's disease	Alzheimer disease	GCST90301303	3x10-12	NA	0.0009881030612147374	PICS	PICS fine-mapped credible set based on reported top hit	STH	0.12393897026777267	2168
B Mem	KANSL1-AS1	rs1378358	17_46751565_G_A	Systemic lupus erythematosus (MTAG)	systemic lupus erythematosus	GCST90270940	1x10-8	-0.066	0.0011557089825039752	PICS	PICS fine-mapped credible set based on reported top hit	MAPT	0.0787731185555458	2416
B Mem	KANSL1-AS1	rs1378358	17_46751565_G_A	Parkinson's disease	Parkinson disease	GCST000528	1x10-14	-0.246860077931525	0.0020996693770410652	PICS	PICS fine-mapped credible set based on reported top hit	MAPT	0.07348910719156265	2278
Mono C	KANSL1-AS1	rs56328224	17_45423221_G_A	Rheumatoid arthritis (PheCode 714.1)	rheumatoid arthritis	GCST90479433	9.100000381469727x10-9	0.009962523781080345	0.00008938737007091834	SuSiE-inf	SuSiE fine-mapped credible set with out-of-sample LD	ARHGAP27	0.4698462188243866	1714
Mono C	KANSL1-AS1	rs56328224	17_45436075_C_T	Systemic lupus erythematosus (MTAG)	systemic lupus erythematosus	GCST90270940	6x10-9	-0.07	0.0002061653130401587	PICS	PICS fine-mapped credible set based on reported top hit	ARHGAP27	0.3517875671386719	2858
Mono C	KANSL1-AS1	rs56328224	17_45673544_C_T	Parkinson's disease	Parkinson disease	GCST90475830	8.974945068359375x10-13	0.009834181096919671	0.00021866866267377706	SuSiE-inf	SuSiE fine-mapped credible set with out-of-sample LD	SPPL2C	0.26543086767196655	2766
Mono C	KANSL1-AS1	rs56328224	17_45628235_A_G	Parkinson's disease	Parkinson disease	GCST003984	4x10-32	0.2407984621529302	0.0002164385650239124	PICS	PICS fine-mapped credible set based on reported top hit	SPPL2C	0.49417245388031006	2470
Mono C	KANSL1-AS1	rs56328224	17_45815607_T_C	Major depressive disorder or hospitalized COVID-19 (pleiotropy)	COVID-19, major depressive disorder	GCST90296444	1x10-12	NA	0.00024699388206306537	PICS	PICS fine-mapped credible set based on reported top hit	STH	0.20836710929870605	2700
Mono C	KANSL1-AS1	rs56328224	17_45826476_C_T	COVID-19 hospitalization or systemic lupus erythematosus (MTAG)	COVID-19, systemic lupus erythematosus	GCST90255371	3x10-18	NA	0.0002408570797569515	PICS	PICS fine-mapped credible set based on reported top hit	SPPL2C	0.23188702762126923	2617
Mono C	KANSL1-AS1	rs56328224	17_45645823_G_A	Ovarian cancer or critical COVID-19 (pleiotropy)	ovarian carcinoma, COVID-19	GCST90296492	8x10-16	NA	0.00025840531171855637	PICS	PICS fine-mapped credible set based on reported top hit	SPPL2C	0.24418936669826508	2629
Mono C	KANSL1-AS1	rs56328224	17_45825631_G_A	Major depressive disorder or severe COVID-19 (pleiotropy)	COVID-19, major depressive disorder	GCST90296443	1x10-9	NA	0.00025722889097326253	PICS	PICS fine-mapped credible set based on reported top hit	STH	0.21132484078407288	2799
Mono C	KANSL1-AS1	rs56328224	17_45834532_C_T	Severe COVID-19 or systemic lupus erythematosus (MTAG)	COVID-19, systemic lupus erythematosus	GCST90255370	2x10-17	NA	0.00022691377914391565	PICS	PICS fine-mapped credible set based on reported top hit	SPPL2C	0.26877960562705994	2602
Mono C	KANSL1-AS1	rs56328224	17_45771384_T_C	Illnesses of mother: Parkinson's disease (UKB data field 20110_11)	Parkinson disease	GCST90042681	6.2085862159729x10-12	-0.0101908146104154	0.0002999149334824875	SuSiE-inf	SuSiE fine-mapped credible set with out-of-sample LD	MAPT	0.2560127377510071	2165
Mono C	KANSL1-AS1	rs56328224	17_45913906_A_G	Alzheimer's disease or educational attainment (pleiotropy)	educational attainment, Alzheimer disease	GCST90095190	7x10-9	-43.902805	0.000264480053375858	PICS	PICS fine-mapped credible set based on reported top hit	MAPT	0.4050951302051544	2846
Mono C	KANSL1-AS1	rs56328224	17_45705327_A_G	Breast cancer or critical COVID-19 (pleiotropy)	COVID-19, breast cancer	GCST90296489	9x10-20	NA	0.0002365661997993494 4	PICS	PICS fine-mapped credible set based on reported top hit	SPPL2C	0.31315359473228455	2575
Mono C	KANSL1-AS1	rs56328224	17_45846317_A_G	Corticobasal degeneration	corticobasal degeneration disorder	GCST002970	1x10-9	1.2412685890696329	0.00025692722839978964	PICS	PICS fine-mapped credible set based on reported top hit	SPPL2C	0.32074519991874695	2800
Mono C	KANSL1-AS1	rs56328224	17_45846317_A_G	Parkinson's disease	Parkinson disease	GCST001126	3x10-14	-0.262364264467491	0.00023872989217533372	PICS	PICS fine-mapped credible set based on reported top hit	SPPL2C	0.30255138874053955	2664
Mono C	KANSL1-AS1	rs56328224	17_45707983_T_C	COVID-19 (hospitalized vs population)	COVID-19	GCST90134597	4x10-12	-0.0943106794712412	0.0002577642085072551	PICS	PICS fine-mapped credible set based on reported top hit	SPPL2C	0.20194369554519653	2708
Mono C	KANSL1-AS1	rs56328224	17_45707983_T_C	COVID-19 severity	COVID-19 symptoms measurement	GCST90274862	5x10-13	-0.0972	0.00025446550225886915	PICS	PICS fine-mapped credible set based on reported top hit	SPPL2C	0.24951985478401184	2685
Mono C	KANSL1-AS1	rs56328224	17_46049399_C_A	Parkinson's disease	Parkinson disease	GCST009512	9x10-13	-0.311323	0.00026558891182343504	PICS	PICS fine-mapped credible set based on reported top hit	MAPT	0.2455509603023529	2745
Mono C	KANSL1-AS1	rs56328224	17_46085231_C_A	COVID-19 (critical illness vs population)	COVID-19	GCST90270934	2x10-16	-0.116533816255951	0.00023155817919422755	PICS	PICS fine-mapped credible set based on reported top hit	SPPL2C	0.15181367099285126	2639
Mono C	KANSL1-AS1	rs56328224	17_46110126_A_G	Parkinson's disease	Parkinson disease	GCST002455	8x10-9	0.44468582126144574	0.00026317386301171006	PICS	PICS fine-mapped credible set based on reported top hit	STH	0.37043941020965576	2859
Mono C	KANSL1-AS1	rs56328224	17_46115202_C_T	Parkinson's disease	Parkinson disease	GCST90455618	1x10-18	-0.25	0.00022448121707618578	PICS	PICS fine-mapped credible set based on reported top hit	STH	0.32601290941238403	2632
Mono C	KANSL1-AS1	rs56328224	17_46142465_T_A	COVID-19 (hospitalized vs population)	COVID-19	GCST90095124	2x10-10	NA	0.0002591717693565732	PICS	PICS fine-mapped credible set based on reported top hit	STH	0.4211447238922119	2786
Mono C	KANSL1-AS1	rs56328224	17_46142465_T_A	COVID-19 with respiratory failure	respiratory failure, COVID-19	GCST90278684	3x10-11	NA	0.00025615423521903113	PICS	PICS fine-mapped credible set based on reported top hit	STH	0.4211447238922119	2756
Mono C	KANSL1-AS1	rs56328224	17_46003698_A_G	Parkinson's disease	Parkinson disease	GCST000855	7x10-12	0.26236426446749106	0.0002476024236949443	PICS	PICS fine-mapped credible set based on reported top hit	MAPT	0.35486161708831787	2725
Mono C	KANSL1-AS1	rs56328224	17_46006036_G_A	Hypothyroidism, strict autoimmune	Hashimoto's thyroiditis	FINNGEN_R12_E4_HYTHY_AI_STRICT	2.3929998874664307x10-8	0.0000902453967742604	0.00064477810676508	SuSie	SuSiE fine-mapped credible set with in-sample LD	MAPT	0.3120294511318207	2647
Mono C	KANSL1-AS1	rs56328224	17_46024409_A_AT	Artrosis, including avohilmo	osteoarthritis	FINNGEN_R12_M13_ARTHROSIS_INCLAVC	3.6589999198913574x10-10	0.00021432375969401 6	0.000162935281890992	SuSie	SuSiE fine-mapped credible set with in-sample LD	MAPT	0.3083437979221344	2451
Mono C	KANSL1-AS1	rs56328224	17_46024409_A_AT	Arthrosis	osteoarthritis	FINNGEN_R12_M13_ARTHROSIS	1.3079999685287476x10-8	0.000871054252696065	0.000172626049284383	SuSie	SuSiE fine-mapped credible set with in-sample LD	MAPT	0.28533899784088135	2336
Mono C	KANSL1-AS1	rs56328224	17_46024483_T_TC	Any dementia (more control exclusions)	Dementia	FINNGEN_R12_KRA_PSY_DEMENTIA_EXM	2.6760001182556152x10-8	-0.0219638173273118	0.0000654596254697628	SuSie	SuSiE fine-mapped credible set with in-sample LD	MAPT	0.20544563233852386	1884
Mono C	KANSL1-AS1	rs56328224	17_45983409_C_T	Breast cancer or hospitalized COVID-19 (pleiotropy)	COVID-19, breast cancer	GCST90296488	4x10-18	NA	0.00021528169364620192	PICS	PICS fine-mapped credible set based on reported top hit	MAPT	0.28448033332824707	2590
Mono C	KANSL1-AS1	rs56328224	17_45983409_C_T	Ovarian cancer or hospitalized COVID-19 (pleiotropy)	ovarian carcinoma, COVID-19	GCST90296491	6x10-16	NA	0.0002245164175502945	PICS	PICS fine-mapped credible set based on reported top hit	MAPT	0.2894947826862335	2624
Mono C	KANSL1-AS1	rs56328224	17_45983409_C_T	COVID-19 (hospitalized covid vs population)	COVID-19	GCST90270935	1x10-18	-0.0943106794712412	0.00021276856115091478	PICS	PICS fine-mapped credible set based on reported top hit	MAPT	0.28448033332824707	2582
Mono C	KANSL1-AS1	rs56328224	17_45983409_C_T	COVID-19 (hospitalized covid vs population)	COVID-19	GCST90454507	1x10-16	-0.0898	0.00022118940133093317	PICS	PICS fine-mapped credible set based on reported top hit	MAPT	0.28448033332824707	2611
Mono C	KANSL1-AS1	rs56328224	17_46152620_T_C	COVID-19 (critical illness vs population or mild symptoms)	COVID-19	GCST90104030	4x10-9	-0.182321556793954	0.0002598978714486131	PICS	PICS fine-mapped credible set based on reported top hit	STH	0.2508043050765991	2833
Mono C	KANSL1-AS1	rs56328224	17_46180422_T_C	Alzheimer's disease polygenic risk score (upper quantile vs lower quantile)	Alzheimer disease, polygenic risk score	GCST90132260	5x10-16	-0.139262067333507	0.00023034921864178203	PICS	PICS fine-mapped credible set based on reported top hit	STH	0.27098676562309265	2630
CD4 ET	KANSL1-AS1	rs55974014	17_45423221_G_A	Rheumatoid arthritis (PheCode 714.1)	rheumatoid arthritis	GCST90479433	9.100000381469727x10-9	0.009962523781080345	0.00005741124167948369	SuSiE-inf	SuSiE fine-mapped credible set with out-of-sample LD	ARHGAP27	0.4698462188243866	1714
CD4 ET	KANSL1-AS1	rs55974014	17_45673544_C_T	Parkinson's disease	Parkinson disease	GCST90475830	8.974945068359375x10-13	0.009834181096919671	0.000020853209619890349	SuSiE-inf	SuSiE fine-mapped credible set with out-of-sample LD	SPPL2C	0.26543086767196655	2766
CD4 ET	KANSL1-AS1	rs55974014	17_45641777_A_G	Parkinson's disease	Parkinson disease	GCST000528	2x10-16	-0.262364264467491	0.000020913456305867196	PICS	PICS fine-mapped credible set based on reported top hit	SPPL2C	0.32658255100250244	2630
CD4 ET	KANSL1-AS1	rs55974014	17_45641777_A_G	Corticobasal degeneration	corticobasal degeneration disorder	GCST002971	1x10-12	1.308332819650179	0.000022717618926197078	PICS	PICS fine-mapped credible set based on reported top hit	SPPL2C	0.32658255100250244	2705
CD4 ET	KANSL1-AS1	rs55974014	17_45815607_T_C	Major depressive disorder or hospitalized COVID-19 (pleiotropy)	COVID-19, major depressive disorder	GCST90296444	1x10-12	NA	0.000020374126756260208	PICS	PICS fine-mapped credible set based on reported top hit	STH	0.20836710929870605	2700
CD4 ET	KANSL1-AS1	rs55974014	17_45645823_G_A	Ovarian cancer or critical COVID-19 (pleiotropy)	ovarian carcinoma, COVID-19	GCST90296492	8x10-16	NA	0.000020443733313407222	PICS	PICS fine-mapped credible set based on reported top hit	SPPL2C	0.24418936669826508	2629
CD4 ET	KANSL1-AS1	rs55974014	17_45825631_G_A	Major depressive disorder or severe COVID-19 (pleiotropy)	COVID-19, major depressive disorder	GCST90296443	1x10-9	NA	0.000022291182509290025	PICS	PICS fine-mapped credible set based on reported top hit	STH	0.21132484078407288	2799
CD4 ET	KANSL1-AS1	rs55974014	17_45771384_T_C	Illnesses of mother: Parkinson's disease (UKB data field 20110_11)	Parkinson disease	GCST90042681	6.2085862159729x10-12	-0.0101908146104154	0.000320926516586163	SuSiE-inf	SuSiE fine-mapped credible set with out-of-sample LD	MAPT	0.2560127377510071	2165
CD4 ET	KANSL1-AS1	rs55974014	17_45913906_A_G	Alzheimer's disease or educational attainment (pleiotropy)	educational attainment, Alzheimer disease	GCST90095190	7x10-9	-43.902805	0.0002220867579296857	PICS	PICS fine-mapped credible set based on reported top hit	MAPT	0.4050951302051544	2846
CD4 ET	KANSL1-AS1	rs55974014	17_45846317_A_G	Corticobasal degeneration	corticobasal degeneration disorder	GCST002970	1x10-9	1.2412685890696329	0.00021974946423159044	PICS	PICS fine-mapped credible set based on reported top hit	SPPL2C	0.32074519991874695	2800
CD4 ET	KANSL1-AS1	rs55974014	17_45707983_T_C	COVID-19 (hospitalized vs population)	COVID-19	GCST90134597	4x10-12	-0.0943106794712412	0.0002191129150504864	PICS	PICS fine-mapped credible set based on reported top hit	SPPL2C	0.20194369554519653	2708
CD4 ET	KANSL1-AS1	rs55974014	17_45707983_T_C	COVID-19 severity	COVID-19 symptoms measurement	GCST90274862	5x10-13	-0.0972	0.00021429110906502394	PICS	PICS fine-mapped credible set based on reported top hit	SPPL2C	0.24951985478401184	2685
CD4 ET	KANSL1-AS1	rs55974014	17_46049399_C_A	Parkinson's disease	Parkinson disease	GCST009512	9x10-13	-0.311323	0.0002068642814015056	PICS	PICS fine-mapped credible set based on reported top hit	MAPT	0.2455509603023529	2745
CD4 ET	KANSL1-AS1	rs55974014	17_46110126_A_G	Parkinson's disease	Parkinson disease	GCST002455	8x10-9	0.44468582126144574	0.00022674201377110683	PICS	PICS fine-mapped credible set based on reported top hit	STH	0.37043941020965576	2859
CD4 ET	KANSL1-AS1	rs55974014	17_46115202_C_T	Parkinson's disease	Parkinson disease	GCST009324	1.0077089071273804x10-12	-0.0418829269594708	0.00023527149029029765	SuSiE-inf	SuSiE fine-mapped credible set with out-of-sample LD	STH	0.3473161458969116	1735
CD4 ET	KANSL1-AS1	rs55974014	17_46142465_T_A	COVID-19 (hospitalized vs population)	COVID-19	GCST90095124	2x10-10	NA	0.00022519187013155418	PICS	PICS fine-mapped credible set based on reported top hit	STH	0.4211447238922119	2786
CD4 ET	KANSL1-AS1	rs55974014	17_46142465_T_A	COVID-19 with respiratory failure	respiratory failure, COVID-19	GCST90278684	3x10-11	NA	0.00022064086599139098	PICS	PICS fine-mapped credible set based on reported top hit	STH	0.4211447238922119	2756
CD4 ET	KANSL1-AS1	rs55974014	17_46003698_A_G	Parkinson's disease	Parkinson disease	GCST000855	7x10-12	0.26236426446749106	0.00020708013233073814	PICS	PICS fine-mapped credible set based on reported top hit	MAPT	0.35486161708831787	2725
CD4 ET	KANSL1-AS1	rs55974014	17_46024409_A_AT	Artrosis, including avohilmo	osteoarthritis	FINNGEN_R12_M13_ARTHROSIS_INCLAVC	3.6589999198913574x10-10	0.00021432375969401 6	0.000165399281433934	SuSie	SuSiE fine-mapped credible set with in-sample LD	MAPT	0.3083437979221344	2451
CD4 ET	KANSL1-AS1	rs55974014	17_46152620_T_C	COVID-19 (critical illness vs population or mild symptoms)	COVID-19	GCST90104030	4x10-9	-0.182321556793954	0.0002247453472361834	PICS	PICS fine-mapped credible set based on reported top hit	STH	0.2508043050765991	2833
CD4 ET	KANSL1-AS1	rs55974014	17_46287153_C_G	Parkinson's disease	Parkinson disease	FINNGEN_R12_G6_PARKINSON	5.935999870300293x10-12	-0.00110625964127491	0.00147610531081006	SuSie	SuSiE fine-mapped credible set with in-sample LD	LRRC37A	0.18633273243904114	2488
CD8 NC	KANSL1-AS1	rs383241	17_45423221_G_A	Rheumatoid arthritis (PheCode 714.1)	rheumatoid arthritis	GCST90479433	9.100000381469727x10-9	0.009962523781080345	0.00006629930350758092	SuSiE-inf	SuSiE fine-mapped credible set with out-of-sample LD	ARHGAP27	0.4698462188243866	1714
CD8 NC	KANSL1-AS1	rs383241	17_45436075_C_T	Systemic lupus erythematosus (MTAG)	systemic lupus erythematosus	GCST90270940	6x10-9	-0.07	0.00022185439559576183	PICS	PICS fine-mapped credible set based on reported top hit	ARHGAP27	0.3517875671386719	2858
CD8 NC	KANSL1-AS1	rs383241	17_45628235_A_G	Parkinson's disease	Parkinson disease	GCST003984	4x10-32	0.2407984621529302	0.000511982036107292 6	PICS	PICS fine-mapped credible set based on reported top hit	SPPL2C	0.49417245388031006	2470
CD8 NC	KANSL1-AS1	rs383241	17_45641777_A_G	Parkinson's disease	Parkinson disease	GCST000528	2x10-16	-0.262364264467491	0.00044008889894170953	PICS	PICS fine-mapped credible set based on reported top hit	SPPL2C	0.32658255100250244	2630
CD8 NC	KANSL1-AS1	rs383241	17_45641777_A_G	Corticobasal degeneration	corticobasal degeneration disorder	GCST002971	1x10-12	1.308332819650179	0.0004193209612472922	PICS	PICS fine-mapped credible set based on reported top hit	SPPL2C	0.32658255100250244	2705
CD8 NC	KANSL1-AS1	rs383241	17_45815607_T_C	Major depressive disorder or hospitalized COVID-19 (pleiotropy)	COVID-19, major depressive disorder	GCST90296444	1x10-12	NA	0.0003845064883169748	PICS	PICS fine-mapped credible set based on reported top hit	STH	0.20836710929870605	2700
CD8 NC	KANSL1-AS1	rs383241	17_45826476_C_T	COVID-19 hospitalization or systemic lupus erythematosus (MTAG)	COVID-19, systemic lupus erythematosus	GCST90255371	3x10-18	NA	0.000392313699239901	PICS	PICS fine-mapped credible set based on reported top hit	SPPL2C	0.23188702762126923	2617
CD8 NC	KANSL1-AS1	rs383241	17_45637484_G_A	Parkinson's disease	Parkinson disease	GCST000959	1x10-28	-0.239016900470499	0.0005255537619457868	PICS	PICS fine-mapped credible set based on reported top hit	SPPL2C	0.3935418725013733	2479

CD8 NC	KANSL1-AS1	rs383241	17_45645823_G_A	Ovarian cancer or critical COVID-19 (pleiotropy)	ovarian carcinoma, COVID-19	GCST90296492	8x10-16	NA	0.00045502986637244923	PICS	PICS fine-mapped credible set based on reported top hit	SPPL2C	0.24418936669826508	2629
CD8 NC	KANSL1-AS1	rs383241	17_45825631_G_A	Major depressive disorder or severe COVID-19 (pleiotropy)	COVID-19, major depressive disorder	GCST90296443	1x10-9	NA	0.0003724526553983766	PICS	PICS fine-mapped credible set based on reported top hit	STH	0.21132484078407288	2799
CD8 NC	KANSL1-AS1	rs383241	17_45834532_C_T	Severe COVID-19 or systemic lupus erythematosus (MTAG)	COVID-19, systemic lupus erythematosus	GCST90255370	2x10-17	NA	0.00040035037447380646	PICS	PICS fine-mapped credible set based on reported top hit	SPPL2C	0.26877960562705994	2602
CD8 NC	KANSL1-AS1	rs383241	17_45771384_T_C	Illnesses of mother: Parkinson's disease (UKB data field 20110_11)	Parkinson disease	GCST90042681	6.2085862159729x10-12	-0.0101908146104154	0.00033230222813518113	SuSiE-inf	SuSiE fine-mapped credible set with out-of-sample LD	MAPT	0.2560127377510071	2165
CD8 NC	KANSL1-AS1	rs383241	17_45913906_A_G	Alzheimer's disease or educational attainment (pleiotropy)	educational attainment, Alzheimer disease	GCST90095190	7x10-9	-43.902805	0.0003581546574699276	PICS	PICS fine-mapped credible set based on reported top hit	MAPT	0.4050951302051544	2846
CD8 NC	KANSL1-AS1	rs383241	17_45917282_C_T	Parkinson's disease	Parkinson disease	GCST004902	1x10-68	-0.248461451298495	0.0004353944538741729	PICS	PICS fine-mapped credible set based on reported top hit	MAPT	0.3641699552536011	2295
CD8 NC	KANSL1-AS1	rs383241	17_45705327_A_G	Breast cancer or critical COVID-19 (pleiotropy)	COVID-19, breast cancer	GCST90296489	9x10-20	NA	0.0004479864787417552	PICS	PICS fine-mapped credible set based on reported top hit	SPPL2C	0.31315359473228455	2575
CD8 NC	KANSL1-AS1	rs383241	17_45917282_C_T	Parkinson's disease	Parkinson disease	GCST002544	2x10-48	-0.262364264467491	0.0004240796396962698	PICS	PICS fine-mapped credible set based on reported top hit	MAPT	0.3641699552536011	2389
CD8 NC	KANSL1-AS1	rs383241	17_45846317_A_G	Corticobasal degeneration	corticobasal degeneration disorder	GCST002970	1x10-9	1.2412685890696329	0.0003715949494509268	PICS	PICS fine-mapped credible set based on reported top hit	SPPL2C	0.32074519991874695	2800
CD8 NC	KANSL1-AS1	rs383241	17_45846317_A_G	Parkinson's disease	Parkinson disease	GCST001126	3x10-14	-0.262364264467491	0.00038934740627344323	PICS	PICS fine-mapped credible set based on reported top hit	SPPL2C	0.30255138874053955	2664
CD8 NC	KANSL1-AS1	rs383241	17_45707983_T_C	COVID-19 (hospitalized vs population)	COVID-19	GCST90134597	4x10-12	-0.0943106794712412	0.0004357470516696504	PICS	PICS fine-mapped credible set based on reported top hit	SPPL2C	0.20194369554519653	2708
CD8 NC	KANSL1-AS1	rs383241	17_45707983_T_C	COVID-19 severity	COVID-19 symptoms measurement	GCST90274862	5x10-13	-0.0972	0.0004422726786371156	PICS	PICS fine-mapped credible set based on reported top hit	SPPL2C	0.24951985478401184	2685
CD8 NC	KANSL1-AS1	rs383241	17_46049399_C_A	Parkinson's disease	Parkinson disease	GCST009512	9x10-13	-0.311323	0.00037536412875949216	PICS	PICS fine-mapped credible set based on reported top hit	MAPT	0.2455509603023529	2745
CD8 NC	KANSL1-AS1	rs383241	17_46085231_C_A	COVID-19 (critical illness vs population)	COVID-19	GCST90270934	2x10-16	-0.116533816255951	0.00037731171486456313	PICS	PICS fine-mapped credible set based on reported top hit	SPPL2C	0.15181367099285126	2639
CD8 NC	KANSL1-AS1	rs383241	17_46110126_A_G	Parkinson's disease	Parkinson disease	GCST002455	8x10-9	0.44468582126144574	0.00035692710439485826	PICS	PICS fine-mapped credible set based on reported top hit	STH	0.37043941020965576	2859
CD8 NC	KANSL1-AS1	rs383241	17_46115202_C_T	Parkinson's disease	Parkinson disease	GCST90455618	1x10-18	-0.25	0.0003798201130614054	PICS	PICS fine-mapped credible set based on reported top hit	STH	0.32601290941238403	2632
CD8 NC	KANSL1-AS1	rs383241	17_46142465_T_A	COVID-19 (hospitalized vs population)	COVID-19	GCST90095124	2x10-10	NA	0.0003767242566868692	PICS	PICS fine-mapped credible set based on reported top hit	STH	0.4211447238922119	2786
CD8 NC	KANSL1-AS1	rs383241	17_46142465_T_A	COVID-19 with respiratory failure	respiratory failure, COVID-19	GCST90278684	3x10-11	NA	0.000380555834613747	PICS	PICS fine-mapped credible set based on reported top hit	STH	0.4211447238922119	2756
CD8 NC	KANSL1-AS1	rs383241	17_46003698_A_G	Parkinson's disease	Parkinson disease	GCST000855	7x10-12	0.26236426446749106	0.0003714421379599747	PICS	PICS fine-mapped credible set based on reported top hit	MAPT	0.35486161708831787	2725
CD8 NC	KANSL1-AS1	rs383241	17_46006036_G_A	Hypothyroidism, strict autoimmune	Hashimoto's thyroiditis	FINNGEN_R12_E4_HYTHY_AI_STRICT	2.3929998874664307x10-8	0.0000902453967742604	0.000394741825583587	SuSie	SuSiE fine-mapped credible set with in-sample LD	MAPT	0.3120294511318207	2647
CD8 NC	KANSL1-AS1	rs383241	17_46024409_A_AT	Arthrosis	osteoarthritis	FINNGEN_R12_M13_ARTHROSIS	1.3079999685287476x10-8	0.000871054252696065	0.000139143416526979	SuSie	SuSiE fine-mapped credible set with in-sample LD	MAPT	0.28533899784088135	2336
CD8 NC	KANSL1-AS1	rs383241	17_45983409_C_T	Breast cancer or hospitalized COVID-19 (pleiotropy)	COVID-19, breast cancer	GCST90296488	4x10-18	NA	0.0003923883087208067	PICS	PICS fine-mapped credible set based on reported top hit	MAPT	0.28448033332824707	2590
CD8 NC	KANSL1-AS1	rs383241	17_45983409_C_T	Ovarian cancer or hospitalized COVID-19 (pleiotropy)	ovarian carcinoma, COVID-19	GCST90296491	6x10-16	NA	0.00038746214106187847	PICS	PICS fine-mapped credible set based on reported top hit	MAPT	0.2894947826862335	2624
CD8 NC	KANSL1-AS1	rs383241	17_45983409_C_T	COVID-19 (hospitalized covid vs population)	COVID-19	GCST90270935	1x10-18	-0.0943106794712412	0.0003936278534701555	PICS	PICS fine-mapped credible set based on reported top hit	MAPT	0.28448033332824707	2582
CD8 NC	KANSL1-AS1	rs383241	17_45983409_C_T	COVID-19 (hospitalized covid vs population)	COVID-19	GCST90454507	1x10-16	-0.0898	0.0003893108663185294	PICS	PICS fine-mapped credible set based on reported top hit	MAPT	0.28448033332824707	2611
CD8 NC	KANSL1-AS1	rs383241	17_46143984_G_A	Lewy body dementia (MTAG)	Lewy body dementia	GCST90133379	4x10-26	-10.5763	0.0004195246309759216	PICS	PICS fine-mapped credible set based on reported top hit	STH	0.42239323258399963	2503
CD8 NC	KANSL1-AS1	rs383241	17_46152620_T_C	COVID-19 (critical illness vs population or mild symptoms)	COVID-19	GCST90104030	4x10-9	-0.182321556793954	0.0003713194871865288	PICS	PICS fine-mapped credible set based on reported top hit	STH	0.2508043050765991	2833
CD8 NC	KANSL1-AS1	rs383241	17_46180422_T_C	Alzheimer's disease polygenic risk score (upper quantile vs lower quantile)	Alzheimer disease, polygenic risk score	GCST90132260	5x10-16	-0.139262067333507	0.00039572135989269564	PICS	PICS fine-mapped credible set based on reported top hit	STH	0.27098676562309265	2630
CD8 NC	KANSL1-AS1	rs383241	17_46287153_C_G	Parkinson's disease	Parkinson disease	FINNGEN_R12_G6_PARKINSON	5.935999870300293x10-12	-0.00110625964127491	0.000631928167483502	SuSie	SuSiE fine-mapped credible set with in-sample LD	LRRC37A	0.18633273243904114	2488
CD4 NC	COX6B1	rs4806187	19_35822162_C_T	Dermatitis and eczema	Eczematoid dermatitis, dermatitis	FINNGEN_R12_L12_DERMATITISECZEMA	1.2869999408721924x10-8	-0.00731600781252292	0.00211396840965711	SuSie	SuSiE fine-mapped credible set with in-sample LD	IGFLR1	0.17542482912540436	76
CD8 ET	COX6B1	rs2285421	19_35822162_C_T	Dermatitis and eczema	Eczematoid dermatitis, dermatitis	FINNGEN_R12_L12_DERMATITISECZEMA	1.2869999408721924x10-8	-0.00731600781252292	0.00147767729353169	SuSie	SuSiE fine-mapped credible set with in-sample LD	IGFLR1	0.17542482912540436	76
B IN	GATS	rs1052482	7_100374211_A_G	Unspecific neurodegenerative disorder	neurodegenerative disease	FINNGEN_R12_G6_NEURODEGENERATIVE	1.4500000476837158x10-8	0.0104371255798952	0.00589088873470151	SuSie	SuSiE fine-mapped credible set with in-sample LD	PILRA	0.2296847254037857	76
B IN	GATS	rs1052482	7_100295525_C_G	Rheumatoid arthritis	rheumatoid arthritis	GCST90132222	2.240999937057495x10-11	0.0867	0.014699486746423674	PICS	PICS fine-mapped credible set extracted from summary sta	GAL3ST4	0.153206929564476	73
B IN	GATS	rs1727134	7_100374211_A_G	Unspecific neurodegenerative disorder	neurodegenerative disease	FINNGEN_R12_G6_NEURODEGENERATIVE	1.4500000476837158x10-8	0.0104371255798952	0.00591676543515286	SuSie	SuSiE fine-mapped credible set with in-sample LD	PILRA	0.2296847254037857	76
B IN	GATS	rs1727134	7_100374211_A_G	Alzheimer's disease (MTAG)	Alzheimer disease	GCST90449062	3.941999912261963x10-9	0.00930636454308	0.001254364940894526	PICS	PICS fine-mapped credible set extracted from summary sta	PILRA	0.378385990858078	39
B IN	GATS	rs1727134	7_100374211_A_G	Alzheimer's disease (late onset)	Alzheimer disease	GCST007319	6x10-9	5.8154516	0.00135048618795799	PICS	PICS fine-mapped credible set based on reported top hit	PILRA	0.38378557562828064	43
B IN	GATS	rs1727134	7_100295525_C_G	Rheumatoid arthritis	rheumatoid arthritis	GCST90132222	2.240999937057495x10-11	0.0867	0.014260783553271693	PICS	PICS fine-mapped credible set extracted from summary sta	GAL3ST4	0.153206929564476	73
B IN	GATS	rs1138417	7_100374211_A_G	Unspecific neurodegenerative disorder	neurodegenerative disease	FINNGEN_R12_G6_NEURODEGENERATIVE	1.4500000476837158x10-8	0.0104371255798952	0.00485671156295954	SuSie	SuSiE fine-mapped credible set with in-sample LD	PILRA	0.2296847254037857	76
B IN	GATS	rs1138417	7_100374211_A_G	Alzheimer's disease (MTAG)	Alzheimer disease	GCST90449062	3.941999912261963x10-9	0.00930636454308	0.0012109188465485089	PICS	PICS fine-mapped credible set extracted from summary sta	PILRA	0.378385990858078	39
B IN	GATS	rs1138417	7_100374211_A_G	Alzheimer's disease (late onset)	Alzheimer disease	GCST007319	6x10-9	5.8154516	0.0013046259816521407	PICS	PICS fine-mapped credible set based on reported top hit	PILRA	0.38378557562828064	43
B IN	GATS	rs1138417	7_100295525_C_G	Rheumatoid arthritis	rheumatoid arthritis	GCST90132222	2.240999937057495x10-11	0.0867	0.014657632403277058	PICS	PICS fine-mapped credible set extracted from summary sta	GAL3ST4	0.153206929564476	73
B IN	GATS	rs1636978	7_100374211_A_G	Unspecific neurodegenerative disorder	neurodegenerative disease	FINNGEN_R12_G6_NEURODEGENERATIVE	1.4500000476837158x10-8	0.0104371255798952	0.00582528801428037	SuSie	SuSiE fine-mapped credible set with in-sample LD	PILRA	0.2296847254037857	76
B IN	GATS	rs1636978	7_100374211_A_G	Alzheimer's disease (MTAG)	Alzheimer disease	GCST90449062	3.941999912261963x10-9	0.00930636454308	0.0012723984090295892	PICS	PICS fine-mapped credible set extracted from summary sta	PILRA	0.378385990858078	39
B IN	GATS	rs1636978	7_100374211_A_G	Alzheimer's disease (late onset)	Alzheimer disease	GCST007319	6x10-9	5.8154516	0.0013695124885920319	PICS	PICS fine-mapped credible set based on reported top hit	PILRA	0.38378557562828064	43
B IN	GATS	rs1636978	7_100295525_C_G	Rheumatoid arthritis	rheumatoid arthritis	GCST90132222	2.240999937057495x10-11	0.0867	0.013599672369637156	PICS	PICS fine-mapped credible set extracted from summary sta	GAL3ST4	0.153206929564476	73
B IN	GATS	rs1636988	7_100374211_A_G	Unspecific neurodegenerative disorder	neurodegenerative disease	FINNGEN_R12_G6_NEURODEGENERATIVE	1.4500000476837158x10-8	0.0104371255798952	0.0058242813799474	SuSie	SuSiE fine-mapped credible set with in-sample LD	PILRA	0.2296847254037857	76
B IN	GATS	rs1636988	7_100374211_A_G	Alzheimer's disease (MTAG)	Alzheimer disease	GCST90449062	3.941999912261963x10-9	0.00930636454308	0.00120908661927307	PICS	PICS fine-mapped credible set extracted from summary sta	PILRA	0.378385990858078	39
B IN	GATS	rs1636988	7_100374211_A_G	Alzheimer's disease (late onset)	Alzheimer disease	GCST007319	6x10-9	5.8154516	0.0013026912439797007	PICS	PICS fine-mapped credible set based on reported top hit	PILRA	0.38378557562828064	43
B IN	GATS	rs1636988	7_100295525_C_G	Rheumatoid arthritis	rheumatoid arthritis	GCST90132222	2.240999937057495x10-11	0.0867	0.011922234665687918	PICS	PICS fine-mapped credible set extracted from summary sta	GAL3ST4	0.153206929564476	73
